# The thromboxane receptor antagonist *NTP42* promotes beneficial adaptation and preserves cardiac function in experimental models of right heart overload

**DOI:** 10.3389/fcvm.2022.1063967

**Published:** 2022-12-14

**Authors:** Eamon P. Mulvaney, Fabiana Renzo, Rui Adão, Emilie Dupre, Lucia Bialesova, Viviana Salvatore, Helen M. Reid, Glória Conceição, Julien Grynblat, Aida Llucià-Valldeperas, Jean-Baptiste Michel, Carmen Brás-Silva, Charles E. Laurent, Luke S. Howard, David Montani, Marc Humbert, Anton Vonk Noordegraaf, Frédéric Perros, Pedro Mendes-Ferreira, B. Therese Kinsella

**Affiliations:** ^1^ATXA Therapeutics Limited, UCD Conway Institute of Biomolecular and Biomedical Research, University College Dublin, Dublin, Ireland; ^2^Department of Surgery and Physiology, Cardiovascular R&D Centre—UnIC@RISE, Faculty of Medicine of the University of Porto, Porto, Portugal; ^3^IPS Therapeutique Inc., Sherbrooke, QC, Canada; ^4^School of Medicine, Université Paris-Saclay, Le Kremlin-Bicêtre, France; ^5^INSERM UMR_S 999, Pulmonary Hypertension: Pathophysiology and Novel Therapies, Hôpital Marie Lannelongue, Le Plessis-Robinson, France; ^6^PHEniX Laboratory, Department of Pulmonary Medicine, Amsterdam UMC (Location VUMC), Amsterdam Cardiovascular Sciences, Vrije Universiteit Amsterdam, Amsterdam, Netherlands; ^7^Amsterdam Cardiovascular Sciences, Pulmonary Hypertension and Thrombosis, Amsterdam, Netherlands; ^8^INSERM UMR_S 1116, Université de Lorraine, Vandoeuvre-lès-Nancy, France; ^9^ToxiPharm Laboratories Inc., Ste-Catherine-de-Hatley, QC, Canada; ^10^Imperial College London, National Heart and Lung Institute, London, United Kingdom; ^11^AP-HP, Dept of Respiratory and Intensive Care Medicine, Pulmonary Hypertension National Referral Centre, Hôpital Bicêtre, Le Kremlin-Bicêtre, France; ^12^Paris-Porto Pulmonary Hypertension Collaborative Laboratory (3PH), INSERM UMR_S 999, Université Paris-Saclay, Le Kremlin-Bicêtre, France; ^13^INSERM, INRAE, CarMeN Laboratory and Centre de Recherche en Nutrition Humaine Rhône-Alpes (CRNH-RA), Claude Bernard University Lyon 1, University of Lyon, Lyon, France; ^14^UCD School of Biomolecular and Biomedical Research, UCD Conway Institute of Biomolecular and Biomedical Research, University College Dublin, Dublin, Ireland

**Keywords:** pulmonary arterial hypertension (PAH), thromboxane receptor, NTP42, right ventricle (RV), heart failure

## Abstract

**Background:**

Pulmonary arterial hypertension (PAH) is a progressive disease characterized by increased pulmonary artery pressure leading to right ventricular (RV) failure. While current PAH therapies improve patient outlook, they show limited benefit in attenuating RV dysfunction. Recent investigations demonstrated that the thromboxane (TX) A_2_ receptor (TP) antagonist *NTP42* attenuates experimental PAH across key hemodynamic parameters in the lungs and heart. This study aimed to validate the efficacy of *NTP42:KVA4*, a novel oral formulation of *NTP42* in clinical development, in preclinical models of PAH while also, critically, investigating its direct effects on RV dysfunction.

**Methods:**

The effects of *NTP42:KVA4* were evaluated in the monocrotaline (MCT) and pulmonary artery banding (PAB) models of PAH and RV dysfunction, respectively, and when compared with leading standard-of-care (SOC) PAH drugs. In addition, the expression of the TP, the target for *NTP42*, was investigated in cardiac tissue from several other related disease models, and from subjects with PAH and dilated cardiomyopathy (DCM).

**Results:**

In the MCT-PAH model, *NTP42:KVA4* alleviated disease-induced changes in cardiopulmonary hemodynamics, pulmonary vascular remodeling, inflammation, and fibrosis, to a similar or greater extent than the PAH SOCs tested. In the PAB model, *NTP42:KVA4* improved RV geometries and contractility, normalized RV stiffness, and significantly increased RV ejection fraction. In both models, *NTP42:KVA4* promoted beneficial RV adaptation, decreasing cellular hypertrophy, and increasing vascularization. Notably, elevated expression of the TP target was observed both in RV tissue from these and related disease models, and in clinical RV specimens of PAH and DCM.

**Conclusion:**

This study shows that, through antagonism of TP signaling, *NTP42:KVA4* attenuates experimental PAH pathophysiology, not only alleviating pulmonary pathologies but also reducing RV remodeling, promoting beneficial hypertrophy, and improving cardiac function. The findings suggest a direct cardioprotective effect for *NTP42:KVA4*, and its potential to be a disease-modifying therapy in PAH and other cardiac conditions.

## Introduction

Pulmonary arterial hypertension (PAH) is a rare yet devastating disease with progressively debilitating symptoms and high mortality. The underlying etiology of PAH is characterized by excessive vasoconstriction and remodeling of the pulmonary vasculature leading to increased pulmonary vascular resistance (PVR). However, the ultimate determinant of survival in PAH patients is the response of the right ventricle (RV) (1, 2).

Upon elevated afterload due to increased PVR, the RV in PAH patients initially responds through compensatory mechanisms termed adaptive hypertrophy. These physiological responses aim to preserve systolic and diastolic right heart function and are characterized by an increased RV wall thickness facilitated by hypertrophic remodeling, increased angiogenesis, altered sarcomere organization and increased intrinsic cardiomyocyte contractility (1, 3, 4). However, in most PAH patients, these adaptive mechanisms are either insufficient or become exhausted, and RV hypertrophy ultimately transitions to pathological maladaptive mechanisms. Maladaptive remodeling of the RV is characterized by a transition to a more eccentric pattern of hypertrophy and a progressive RV dilation resulting in a leftward septal shift impacting left ventricular (LV) function (5, 6). In addition, the consequent elevation in RV wall tension results in an increased metabolic demand and a simultaneous decrease in myocardial perfusion capacity, leading to decreased RV contractility despite progressive increases in afterload (7). Furthermore, RV diastolic function is compromised through increased stiffness primarily due to cardiomyocyte hypertrophy and fibrosis. In PAH patients, all these factors contribute to progressive right heart dysfunction, ultimately resulting in heart failure.

While considerable advances have been made in the clinical management of PAH, patient mortality remains high. Current PAH standard-of-care (SOC) therapies include the phosphodiesterase type-5 inhibitors (PDE5is), endothelin receptor antagonists (ERAs), prostacyclin analogs (PCAs) or prostacyclin receptor agonists (PRAs) and soluble guanylate cyclase (sGC) stimulators, with various other pipeline compounds in clinical development. The key focus of the SOCs and those in clinical development is to reduce PVR, either by alleviating excessive pulmonary vasoconstriction or enhancing pulmonary vasodilation, and/or reduce pulmonary vascular remodeling. However, while it is recognized that RV function is the main determinant of prognosis in PAH, current SOC therapies have limited cardiac-specific effects (8, 9). Furthermore, in PAH there is a paradox where RV function continues to deteriorate and have poorer survival outcomes despite reductions in PVR observed using current PAH SOC therapies (10). Consequently, there has been a shift in the clinical thinking from solely considering the effects of PAH therapies on PVR to instead investigating their potential for directly addressing the effects on the RV.

The thromboxane (TX) A_2_ receptor, or TP, primarily mediates signaling of the prostanoid TXA_2_ and of the free-radical derived isoprostane 8-iso-prostaglandin F_2α_ (8-iso-PGF_2α_), as well as other endogenous ligands, regulating processes including platelet aggregation, and constriction and proliferation of vascular and pulmonary smooth muscle. TP-mediated signaling also mediates potent *pro*-inflammatory, *pro*-mitogenic, and *pro*-fibrotic effects, and levels of TXA_2_, 8-iso-PGF_2α_, and TP expression are elevated in many cardiovascular and pulmonary diseases, inflammatory disorders and in certain cancers (11, 12). Multiple studies have shown the importance of TP signaling in the development and progression of PAH (13–19). TXA_2_ mimetics induce ventricular arrhythmia, and TP signaling contributes to cardiac hypertrophy and fibrosis in multiple animal models (19–26). TP expression has been demonstrated to be specifically elevated in certain pathological cardiac conditions, and both TP receptor occupancy and expression is elevated in the RV of PAH patients compared to non-diseased subjects (18, 19). In addition, the TXA_2_/TP signaling axis contributes to cardiac hypertrophy in multiple animal models of systemic hypertension (24, 25). While activation of the TP is profibrotic in multiple systems, including within the heart, TP antagonism with CPI211 (Ifetroban) decreased RV fibrosis and improved cardiac function in a pulmonary artery banding (PAB) model of RV pressure overload (19). Furthermore, TP antagonism improved cardiac output, increased ejection fraction while decreasing cardiac fibrosis and transforming growth factor (TGF)−β signaling in mouse models of Duchenne muscular dystrophy (DMD) (26). Taken together, these studies suggest a broader pattern of deleterious consequences of TP activation on the heart.

The TP antagonist *NTP42* is currently in clinical development for PAH and other cardiopulmonary indications. Previous efficacy evaluations demonstrated that *NTP42* attenuates preclinical PAH in both the monocrotaline (MCT)-and Sugen/Hypoxia (SuHx)-induced animal models of PAH (27, 28). As a drug specifically developed as an oral formulation for clinical use, *NTP42:KVA4* was recently evaluated in a randomized, placebo-controlled first-in-human Phase I clinical trial (NCT04919863) in 79 healthy male volunteers where it was confirmed as safe, well-tolerated, with good pharmacokinetic and pharmacodynamic profiles following single and repeat oral dosing. To specifically assess its potential to impart direct cardioprotective effects on the RV, the aim of this study was to validate the efficacy of *NTP42*, delivered as the orally formulated *NTP42:KVA4*, in the MCT-PAH model while also exploring its effect in the pulmonary artery banding (PAB) preclinical model of RV dysfunction and pressure overload. Furthermore, in this study, we also examined expression levels of the TP, the target receptor for *NTP42*, in RV tissues from several highly relevant disease models as well as in clinical specimens from subjects with PAH and dilated cardiomyopathy (DCM), where the data further supports the hypothesis that the TP is a *bona fide* target for treatment of PAH and certain other cardiac dysfunctions.

## Materials and methods

### Animal models

All experiments were carried out in accordance with US NIH guidelines. Male Sprague-Dawley rats (Charles River Laboratories) were used in all models. MCT-PAH was induced using a single injection of 60 mg/kg MCT, where twice-daily oral treatment with placebo, *NTP42:KVA4* (1 mg/kg), the PDE5i Sildenafil (50 mg/kg), the ERA Macitentan (30 mg/kg), the PRA Selexipag (1 mg/kg), or the sGC stimulator Riociguat (5 mg/kg) was commenced on Day 7 post-MCT and continued to Day 28 (Supplementary material). The PAB model used surgical banding of the pulmonary artery, where twice-daily oral treatment with placebo, *NTP42:KVA4* (1 mg/kg), or the sGC stimulator Riociguat (5 mg/kg) was started on Day 2 post-PAB and continued to Day 27 (Supplementary material).

### Human tissues

Human tissues, following autopsy, were obtained from the Institute of Cardiometabolism and Nutrition BioCollection (Paris, France), detailed in Supplementary Table 2. Protocols to obtain human biospecimens conformed with the recommendations of the Declaration of Helsinki.

A detailed description of the Materials and methods is presented in Supplementary material. Detailed descriptions of all materials and methods used in this study, including chemicals, animals and surgical procedures, tissue harvesting, preparation and histological staining and analysis, isolated cardiomyocyte force transduction experiments, quantitative real-time PCR, Western blotting, and statistical analyses are presented in Supplementary material. Statistical methods are also summarized in each figure legend, where values are expressed as mean ± standard error of the mean (SEM) and number of replicates (n). Throughout, *P*-values < 0.05 were considered to indicate significant differences.

## Results

### *NTP42:KVA4* improves right ventricular adaptation in the monocrotaline- pulmonary arterial hypertension model

We previously reported that *NTP42* alleviates pulmonary pathologies and cardiopulmonary hemodynamics in both the MCT- and SuHx-induced PAH animal models (27, 28). In those studies, *NTP42* was administered orally as the active pharmaceutical ingredient (API) following its dissolution in an organic-based drug vehicle unsuited and not approved for use in man. Hence, for use in the clinical setting, *NTP42* has since been uniquely formulated with the widely used pharmaceutical polymer Kollidon^®^ VA 64 yielding the investigational medicinal product (IMP) referred to as *NTP42:KVA4*. Herein, it was first necessary to confirm or validate the oral efficacy of *NTP42* administered as *NTP42:KVA4* in preclinical PAH. Thereafter, the study specifically aimed to investigate the preclinical efficacy of *NTP42:KVA4* in the PAB model of RV pressure overload to assess the potential of *NTP42* to impart direct cardiac benefits.

Hence, efficacy of *NTP42:KVA4* was first evaluated in a delayed interventional MCT-PAH model in rodents, where disease was allowed to develop for 7 days prior to initiating treatment, where efficacy was also compared with drugs from each of the four clinical SOC PAH therapies.

**FIGURE 1:**
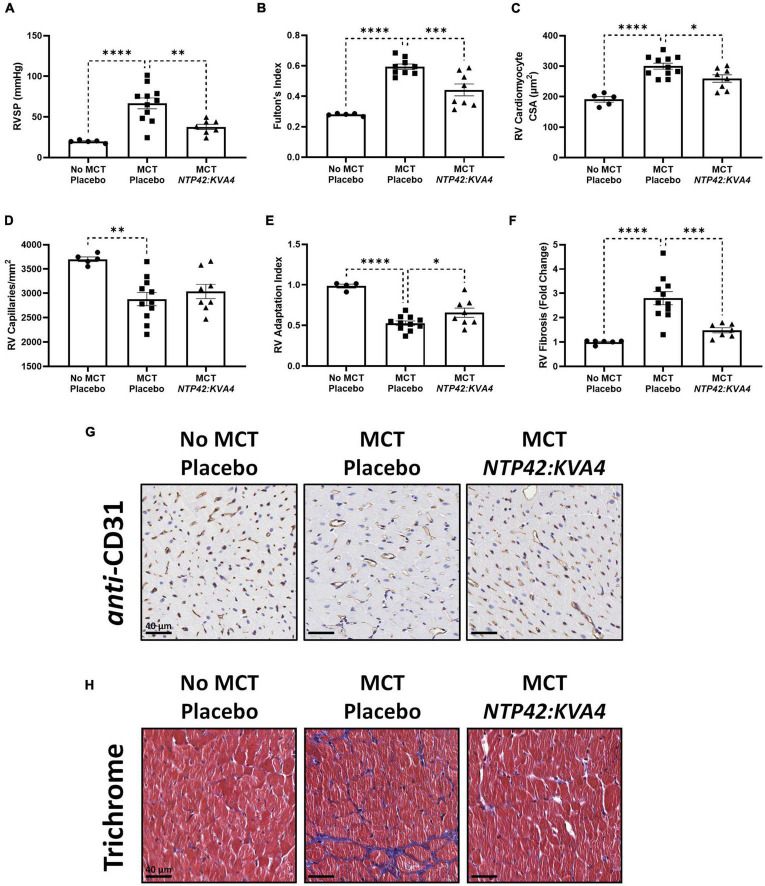
Effect of *NTP42:KVA4* treatment on RV hypertrophy and pathology in the MCT-PAH model. Normal control rats (“No MCT—Placebo”) and rats injected with MCT (60 mg/kg) were treated twice daily orally (PO BID) with either drug placebo (“MCT—Placebo”) or *NTP42:KVA4* (1 mg/kg PO BID), starting from Day 7 following administration of MCT. **(A–F)** show: **(A)** RVSP in the “No MCT—Placebo,” “MCT—Placebo,” and *NTP42:KVA4* groups [*n* = 5, 11, 7], **(B)** Fulton’s Index [*n* = 5, 9, and 8, respectively]; **(C)** RV cardiomyocyte size [*n* = 5, 11, 8]; **(D)** RV vascularization [*n* = 5, 11, 8]; **(E)** RV adaptation index [*n* = 6, 11, 8], and **(F)** RV fibrosis [*n* = 5, 11, 7]. Data presented are the mean ± SEM. *P < 0.05, **P < 0.01, ***P < 0.001, ****P < 0.0001 vs. “MCT—Placebo,” according to one-way analysis of variance (ANOVA) with Holm-Šídák correction applied for multiple comparisons. **(G,H)** Show representative photomicrographs, selected from a random animal from each treatment group, of: **(G)** *Anti*-CD31-stained RV tissue captured at 200 × magnification (scale bars represent 40 μm), and **(H)** Masson’s trichrome-stained RV tissue captured at 200 × magnification (scale bars, 40 μm). Note that while Supplementary Table 1 provides details on numbers of animals enrolled into the studies reported herein and those that survived through to terminal surgery, the numbers (n) given in the square brackets in all figure legends refer to the number of input data used for the individual experimental parameter following removal of any justifiable outliers identified using the method of Interquartile Range with Tukey fences.

MCT led to increased RV systolic pressure (RVSP) and RV hypertrophy, as measured by Fulton’s Index (Figures 1A,B). Quantification of cardiomyocyte size, measured as cross-sectional area at the cellular level, and vascularization demonstrated that this RV hypertrophy was typical of maladaptive processes. Specifically, RV cardiomyocytes from MCT-treated animals were significantly enlarged, while RV capillary density was significantly reduced which, combined, resulted in a decrease in the RV Adaptation Index, the ratio of vascularization to cardiomyocyte size (Figures 1C–E,G). Consistent with the myocardial disorganization that occurs in abnormal hypertrophic processes, Masson’s trichrome staining also revealed pronounced fibrosis in the RV of MCT-treated animals (Figures 1F,H). Treatment with *NTP42:KVA4* significantly alleviated the MCT-induced increases in RVSP and Fulton’s Index parameters and alleviated the MCT-induced increase in cardiomyocyte size (Figures 1A–C). While *NTP42:KVA4* did not significantly affect RV vascularization in this PAH model *per se*, it did result in an improvement in the RV Adaptation Index (Figures 1D–E,G). Moreover, *NTP42:KVA4* significantly attenuated MCT-induced RV fibrosis (Figures 1F,H). In line with previous investigations,(27) treatment with *NTP42:KVA4* also significantly alleviated the MCT-induced increase in mean pulmonary arterial pressure (mPAP), and significantly attenuated pulmonary pathologies, including vessel occlusion and muscularization, CD68^+^ macrophage infiltration, perivascular fibrosis, and edema (Supplementary Figure 1).

Treatment with the PAH SOCs Sildenafil, Macitentan and Riociguat, but not with Selexipag, also resulted in decreased mPAP, RVSP and Fulton’s Index (Supplementary Table 4). In contrast to *NTP42:KVA4*, none of these SOCs significantly reduced cardiomyocyte size (Supplementary Table 4). Sildenafil alone resulted in increased RV vascularization while Sildenafil, Macitentan, and Riociguat, but not Selexipag, significantly increased the RV Adaptation Index and decreased the levels of RV fibrosis (Supplementary Table 4). While the PAH SOCs decreased vessel occlusion and muscularization as well as CD68^+^ macrophage infiltration (Supplementary Table 4), they did not significantly reduce perivascular fibrosis or edema (Supplementary Table 4). Notably, while neither *NTP42:KVA4*, Sildenafil or Macitentan affected mean systemic arterial pressure (mAP) or heart rate (HR) *per se*, animals treated with Riociguat or Selexipag displayed either increased mAP or decreased HR, respectively (Supplementary Table 4).

### *NTP42:KVA4* attenuates right ventricular structural changes and dysfunction in the pulmonary artery banding model

Following validation of the efficacy of the formulated *NTP42:KVA4* in the MCT-PAH model, the potential for a direct benefit of TP antagonism on cardiac adaptation and function was investigated using the PAB model of RV pressure overload. Notably, while RV hypertrophy and dysfunction are features of the MCT-PAH model, and findings of cardiac benefits for therapeutic agents in this model are indeed valuable, the MCT-PAH model has two important limitations in this regard (29). Firstly, as the RV and the pulmonary vasculature are functionally coupled, direct cardiac-specific effects for an interventional therapy cannot be readily distinguished from afterload reductions due any benefits of the therapy on the pulmonary vasculature, i.e., its pulmonary-specific effects. In addition, the toxic MCT alkaloid itself may have direct effects on the RV, inducing confounding pathologies including myocarditis and arrythmias (30). The use of the PAB model herein to induce a chronic pressure load on the RV aimed to circumvent these limitations. Treatment with *NTP42:KVA4* (1 mg/kg PO BID) or Riociguat (5 mg/kg PO, BID) was initiated 2 days after PAB surgery, where Riociguat was chosen as an appropriate comparator compound as, in previous preclinical investigations, it has been shown to prevent deterioration of RV function induced by PAB (31). In addition, Riociguat is the only PAH SOC which has demonstrated potential clinical uses in heart failure settings, (32) where other SOC compounds have demonstrated conflicting findings, often with adverse outcomes (33, 34). Pre-treatment echocardiogram (ECHO) showed robust and comparable pulmonary arterial (PA) pressure gradients across the randomized PAB animal groups (Supplementary Figures 2A,B). At study termination, animals subjected to PAB showed comparable bodyweight and no differences in bodyweight gain over the course of the study was observed (Supplementary Figures 2C,D).

**FIGURE 2:**
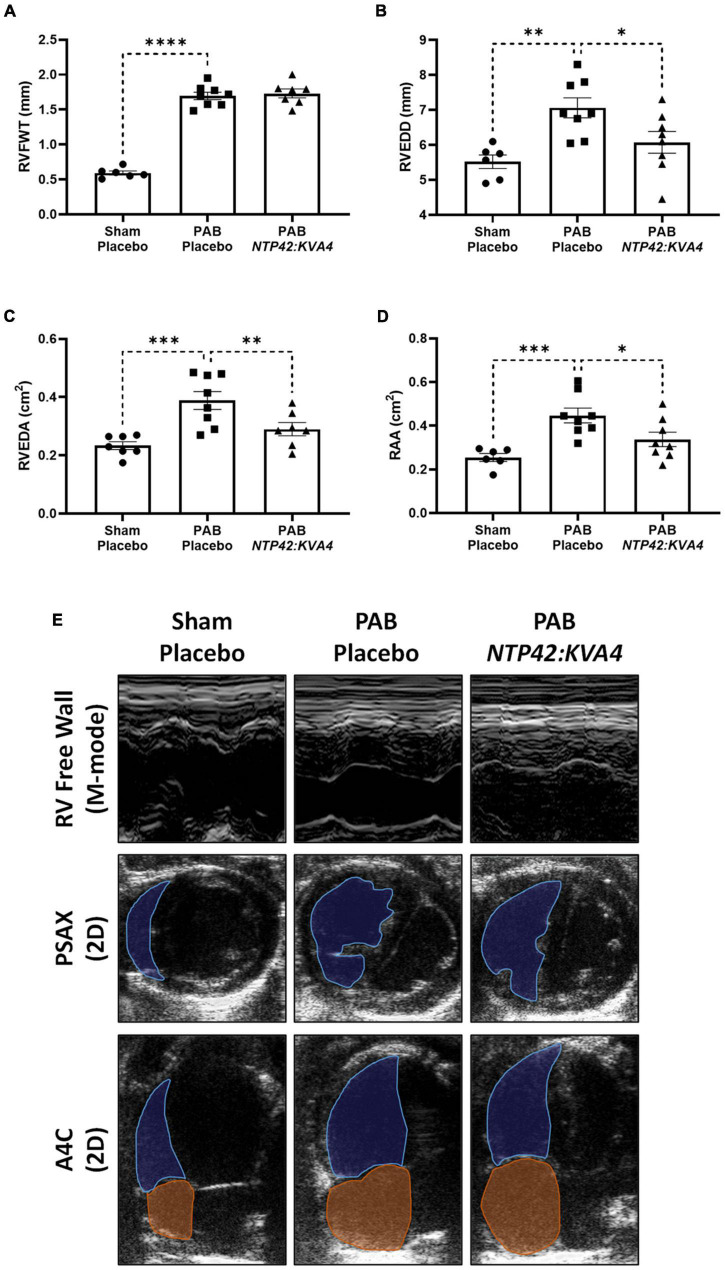
Effect of *NTP42:KVA4* treatment on right heart dimensions and geometry in the PAB model. Normal control rats (“Sham—Placebo”) and rats subjected to pulmonary arterial banding (PAB) were treated with either drug placebo (“PAB—Placebo”) or *NTP42:KVA4* (1 mg/kg PO BID), starting from Day 2 following PAB. **(A–D)** Show ECHO-derived measurements of: **(A)** RVFWT in the “Sham—Placebo,” “PAB—Placebo,” and *NTP42:KVA4* groups [*n* = 6, 8, and 7, respectively]; **(B)** RVEDD [*n* = 6, 8, 8]; **(C)** RVEDA [*n* = 7, 8, 7], and **(D)** RA area (RAA) [*n* = 6, 8, 7]. Data presented are the mean ± SEM. *P < 0.05, **P < 0.01, ***P < 0.001, ****P < 0.0001 vs. “PAB—Placebo,” according to one-way ANOVA with Holm-Šídák correction. **(E)** Shows representative ECHO images from M-mode recordings and from 2D parasternal short-axis (PSAX) and apical four-chamber (A4C) views selected from a random animal from each treatment group and where blue and orange shading and lines delineate the RV and RA, respectively.

In ECHO assessments, PAB resulted in increased RV free wall thickness (RVFWT) and RV dilation as evidenced by increased RV end-diastolic dimension (RVEDD) and RV end-diastolic area (RVEDA) (Figures 2A–C). In addition, the right atrial area (RAA) was enlarged (Figure 2D). Treatment with *NTP42:KVA4* improved RV geometries and attenuated RV dilation, where both RVEDD and RVEDA were reduced (Figures 2B,C). Notably, the attenuated RV dilation observed following *NTP42:KVA4* treatment was not paralleled by compromised RV hypertrophy, where RVFWT was unchanged (Figure 2A). In addition, *NTP42:KVA4* alleviated PAB-induced RAA enlargement (Figure 2D). Comparable benefits on right heart geometry were not observed following treatment with Riociguat (Supplementary Table 5).

**FIGURE 3:**
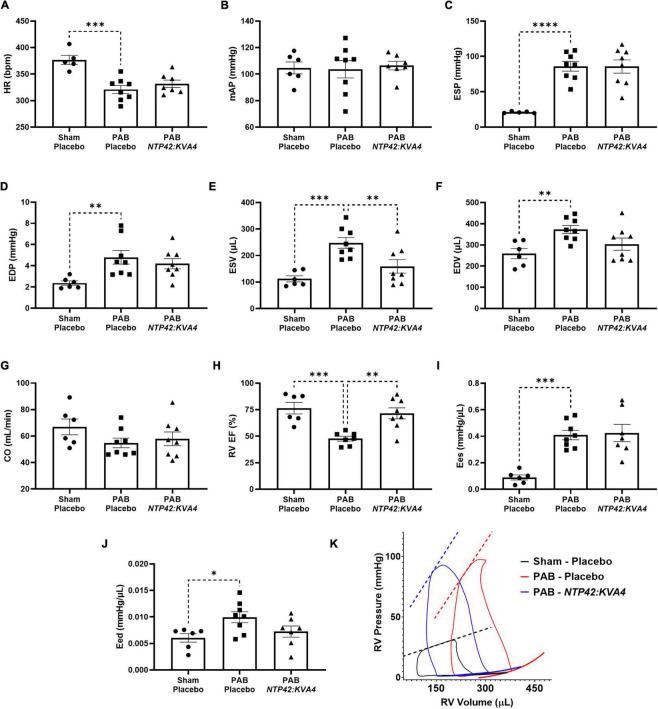
Effect of *NTP42:KVA4* treatment on RV pressure, volume and function in the PAB model. Hemodynamic measurements of: **(A)** HR in the “Sham—Placebo,” “PAB—Placebo,” and *NTP42:KVA4* groups [*n* = 5, 8, and 7, respectively]; **(B)** mAP [*n* = 6, 8, 7]; **(C)** RV ESP [*n* = 5, 8, 8]; **(D)** RV EDP [*n* = 6, 8, 8]; **(E)** RV ESV [*n* = 6, 8, 8]; **(F)** RV EDV [*n* = 6, 8, 8]; **(G)** CO [*n* = 6, 8, 8]; **(H)** RV EF [*n* = 6, 7, 8]; **(I)** RV Ees [*n* = 6, 8, 7], and **(J)** RV Eed [*n* = 6, 8, 7]. Data presented are the mean ± SEM. *P < 0.05, **P < 0.01, ***P < 0.001, ****P < 0.0001 vs. “PAB—Placebo,” according to one-way ANOVA with Holm-Šídák correction. **(K)** shows representative RV PV loops from PAB study animals. The linear end-systolic and exponential end-diastolic PV relationships within each group are displayed as thick dashed or solid lines, respectively. Maximum/minimum PV points on the displayed representative loops, and the PV relationships plotted thereon, were adjusted to correspond approximately with the average values determined within treatment group.

Detailed pressure-volume (PV) loop analyses showed that PAB animals demonstrated profound RV overload with marked signs of RV dysfunction (Figure 3). In PAB animals, HR was decreased with unchanged mAP (Figures 3A,B), and RV end-systolic pressure (ESP) was fourfold higher than in Sham animals (Figure 3C). RV filling pressure (end diastolic pressure, EDP) was also increased in PAB animals (Figure 3D), and RV dilation was observed when considering both end-systolic and end-diastolic volumes (ESV and EDV; Figures 3E,F). While cardiac output (CO) was reduced upon PAB (–18%, *P* = 0.1998, Figure 3G), the RV ejection fraction (RV EF) was significantly compromised relative to the Sham control (Figure 3H). End-systolic elastance (Ees) was increased in PAB animals (Figure 3I), and significant diastolic dysfunction was evident in this group, as demonstrated by increased end-diastolic elastance (Eed) (Figure 3J).

In this model, treatment with *NTP42:KVA4* significantly improved RV function. In line with ECHO data (Figure 2), *NTP42:KVA4* markedly reduced RV dilation, where both ESV and EDV were reduced relative to the PAB control (Figures 3E,F). Most notably, *NTP42:KVA4* significantly improved RV EF relative to PAB control (Figure 3H), resulting in near-normalized values compared with Sham animals. In addition, *NTP42:KVA4* trended toward an improvement (27%, *P* = 0.0641) in diastolic function, as measured by Eed (Figure 3J).

While treatment with Riociguat led to improvements in measures of RV dilation, and increased RV EF, albeit to a lesser extent than *NTP42:KVA4*, Riociguat treatment trended toward a further worsening in Eed (24%, *P* = 0.2908; Supplementary Table 5).

### *NTP42:KVA4* promotes an adaptive pattern of right ventricular hypertrophy and reduces expression of genes associated with cardiac dysfunction in the pulmonary artery banding model

**FIGURE 4:**
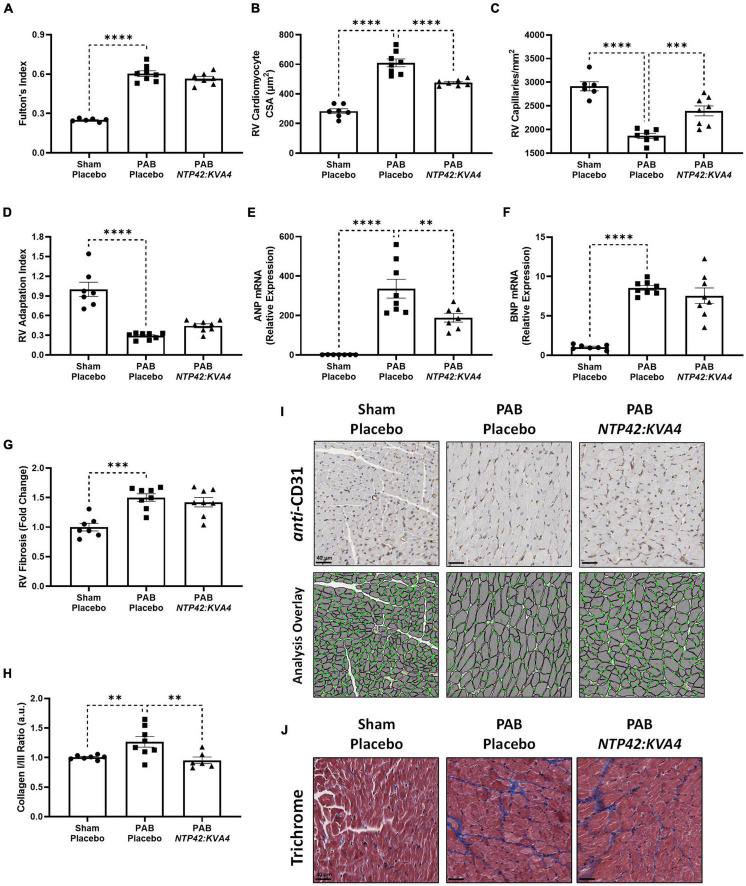
Effect of *NTP42:KVA4* treatment on RV hypertrophy and pathology in the PAB model. **(A–H)** show: **(A)** Fulton’s Index in the “Sham—Placebo,” “PAB—Placebo,” and *NTP42:KVA4* groups [*n* = 6, 8, and 7, respectively]; **(B)** RV cardiomyocyte size [*n* = 7, 8, 7]; **(C)** RV vascularization [*n* = 6, 7, 8]; **(D)** RV adaptation index [*n* = 7, 8, 8], **(E)** RV ANP mRNA expression [*n* = 7, 8, 7], **(F)** RV BNP mRNA expression [*n* = 7, 8, 8], **(G)** RV fibrosis [*n* = 7, 8, 8], and **(H)** RV collagen type I/III ratio, calculated from the RV collagen type I and III mRNA expression for each animal, and expressing the ratio in arbitrary units (a.u.) [*n* = 7, 8, 6]. Data presented are the mean ± SEM. **P < 0.01, ***P < 0.001, ****P < 0.0001 vs. “PAB—Placebo,” according to one-way ANOVA with Holm-Šídák correction. **(I,J)** show representative photomicrographs, selected from a random animal from each treatment group, of: **(I)** (Upper panel): *Anti*-CD31-stained RV tissue captured at 400 × magnification (scale bars represent 40 μm), (Lower panel): Image analysis overlay showing annotated cardiomyocyte cross-sectional area (gray outline) and CD31^+^ vessels (green dots), and **(J)** Masson’s trichrome-stained RV tissue captured at 400 × magnification (scale bars, 40 μm).

While *NTP42:KVA4* treatment did not lead to reductions in the gross RV wall enlargement induced by PAB (Fulton’s Index, Figure 4A), it significantly decreased cardiomyocyte size relative to PAB controls (Figures 4B,I), consistent with findings from the MCT-PAH model (Figure 1C). In addition, in this PAB model, *NTP42:KVA4* treatment significantly increased RV vascularization (Figures 4C,I). Considering both factors, *NTP42:KVA4* treatment resulted in a trend toward improvement in the RV Adaptation Index (*P* = 0.0797; Figure 4D). Furthermore, expression analysis of genes associated with RV hypertrophy showed that levels of atrial natriuretic peptide (ANP) and brain natriuretic peptide (BNP) were significantly increased upon PAB, relative to Sham levels (Figures 4E,F). Treatment with *NTP42:KVA4* significantly reduced ANP levels (Figure 4E) and trended toward reduced BNP levels (Figure 4F).

Notably, RV fibrosis, while increased upon PAB (Figures 4G,J), was less pronounced than that observed in the MCT-PAH model (Figure 1G), and reductions in this gross level of fibrosis were not observed with *NTP42:KVA4* in the PAB model. Notably, fibrosis-mediated myocardial stiffness is influenced by the predominant collagen isoform, where an increased ratio of the stiff type I isoform relative to the elastic type III isoform is linked with more severe RV dysfunction (35). Herein, analysis of collagen isoform expression levels demonstrated a marked increase in the collagen I/III ratio upon PAB (Figure 4H). Treatment with *NTP42:KVA4* significantly reduced the collagen I/III ratio (Figure 4H), indicative of predominant expression of the more flexible collagen III isoform.

In this model, total heart weight was significantly increased in all PAB groups (Supplementary Figure 3A). Consistent with ECHO assessments showing RA chamber enlargement (Figure 2D), measurements of the RA wall weight demonstrated that substantial RA remodeling occurred in response to PAB, and where *NTP42:KVA4* treatment led to significant reductions in this index (Supplementary Figure 3B). Notably, while PAB did not induce changes in left ventricular (LV) cardiomyocyte size or vascularization, an increased level of LV fibrosis was appreciable which was somewhat reduced by *NTP42:KVA4* treatment (–28%, *P* = 0.1386, Supplementary Figure 3C–G).

In these analyses, the PAH SOC Riociguat did not lead to significant improvements in Fulton’s Index, cardiomyocyte size, RV vascularization, ANP mRNA expression levels, or RV fibrosis, and an improved pattern of adaptive hypertrophy was not observed upon Riociguat treatment (Supplementary Table 5). However, like *NTP42:KVA4*, Riociguat led to a reduction in the collagen I/III ratio (Supplementary Table 5).

### *NTP42:KVA4* results in normalized cardiomyocyte passive tension and reduces proteolytic degradation of calcium-handling proteins in the pulmonary artery banding model

**FIGURE 5:**
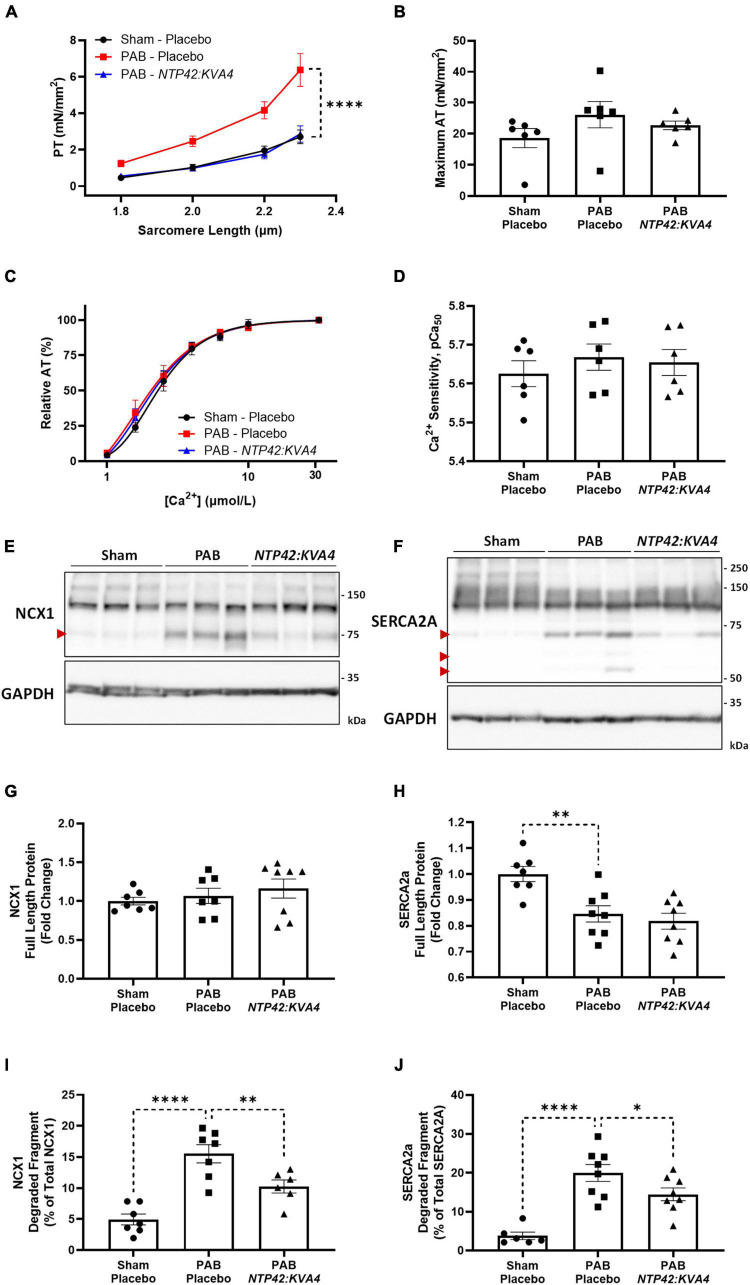
Effect of *NTP42:KVA4* treatment on isolated cardiomyocyte force transduction and calcium-handling protein expression in the PAB model. **(A–D)** show: **(A)** Steady-state PT measured at increasing sarcomere lengths (1.8–2.3 μm) from cardiomyocytes isolated from animals in the “Sham—Placebo,” “PAB—Placebo,” and *NTP42:KVA4* groups [*n* = 6, 6, and 6, respectively]; **(B)** Maximum AT development at [Ca^2+^] 31.6 μmol/L [*n* = 6, 6, 6]; **(C)** Relative AT development in response to increasing submaximal free Ca^2+^ concentration ([Ca^2+^], 1–31.6 μmol/L) [*n* = 6, 6, 6], where calcium response curves were fitted using non-linear regression, and **(D)** Calcium sensitivity (pCa_50_) determined from individual regression analyses [*n* = 6, 6, 6]. **(E,F)** Show western blot protein expression of: **(E)** NCX1 and **(F)** SERCA2a, where representative RV lysates are displayed from 3 random animals from each treatment group and glyceraldehyde-3-phosphate dehydrogenase (GAPDH) expression levels were used as loading control. The relative positions of the molecular size markers (kDa) are indicated to the right of the panels and the positions of the observed degraded fragments of NCX1 and SERCA2a are marked with red arrowheads to the left of the panels. **(G–J)** Show mean relative RV expression levels of **(G)** NCX1 full-length protein (120 kDa) [*n* = 7, 7, 8]; **(H)** SERCA2a full-length protein (110 kDa) [*n* = 7, 8, 8]; **(I)** NCX1 75 kDa degraded fragment, expressed as a percentage of total NCX1 [*n* = 7, 7, 6], and **(J)** SERCA2a 70 kDa degraded fragment, expressed as a percentage of total SERCA2a [*n* = 6, 8, 8]. Data presented are the mean ± SEM, and where in **(A–D)**, results are presented from 6 animals per group, with an average of 5 independent cardiomyocytes (technical replicates) analyzed per animal. *P < 0.05, **P < 0.01, ****P < 0.0001 vs. “PAB—Placebo,” according to two-way ANOVA with Holm-Šídák correction **(A,C)** or one-way ANOVA with Holm-Šídák correction **(B,D,G–J)**.

While PAB-induced right heart pressure overload resulted in significant structural remodeling and systolic and diastolic dysfunction, intrinsic changes in cardiomyocyte tension development were also observed in this model. Passive tension (PT) was significantly increased across all sarcomere lengths in PAB animals, indicative of increased cardiomyocyte stiffness (Figure 5A). Consistent with the improvement in diastolic function observed for *NTP42:KVA4* (Figure 3J), PT was significantly attenuated in cardiomyocytes isolated from *NTP42:KVA4*-treated animals (Figure 5A) and was indistinguishable from the profile of Sham control animals. Regarding systolic function, cardiomyocytes isolated from PAB control animals showed somewhat increased maximum active tension (AT) compared with Sham animals (Figure 5B), while relative AT profiles were similar between Sham, PAB and *NTP42:KVA4* groups (Figure 5C). Similar benefits on intrinsic cardiomyocyte function were not observed following Riociguat treatment in this model and both PT and AT were increased in comparison with the PAB control (Supplementary Table 5).

Notably, despite the altered profiles of contraction, no changes in calcium sensitivity were observed between the groups (Figure 5D). Besides altered calcium sensitivity, a further mechanism contributing to cardiomyocyte dysfunction involves decreased capacity for diastolic calcium clearance (36). For efficient cardiomyocyte relaxation, cytosolic calcium levels must promptly drop following contraction, where this is facilitated either by its efflux from the cell by Na^+^/Ca^2+^ exchanger 1 (NCX1) or sequestration into internal cellular stores by sarco/endoplasmic reticulum Ca^2+^-ATPase 2a (SERCA2a) (37). Alterations in the expression of these calcium-handling proteins contribute to cardiomyocyte dysfunction during pressure-induced hypertrophy and cardiac failure (38–40), and decreases in NCX1 and SERCA2a have been observed in the RV from PAH patients (36). Furthermore, degradation and inactivation of NCX1 and SERCA2a, mediated by the calcium-activated protease calpain, occurs in multiple animal models of heart failure (41–44). Herein, while expression of intact full-length NCX1 was unchanged, a significant decrease in SERCA2a protein was observed in PAB animals (Figures 5E–H). Moreover, elevated degradation of both NCX1 and SERCA2a were observed following PAB (Figures 5E,F,I,J). Specifically, increased expression of a single degradation fragment of NCX-1 and up to three SERCA2a degradation fragments were observed (Figures 5E,F), where these inactive fragments have been previously described (41, 44). Treatment with *NTP42:KVA4*, but not Riociguat, attenuated calcium-handling protein degradation, with significantly reduced NCX1 and SERCA2a degradation fragments evident (Figures 5E,F,I,J).

#### Thromboxane receptor expression is elevated in the right ventricle in experimental models and in human pulmonary arterial hypertension and other right ventricular conditions

While signaling through the TP is implicated in pathological cardiac conditions, few studies have examined TP expression levels in RVs of subjects with PAH. Thus, as a rationale for the therapeutic potential and utility of TP antagonists *per se*, expression of the TP was examined in RV tissues from experimental PAH and cardiac disease models, as well as in clinical specimens of PAH and dilated cardiomyopathy (DCM).

**FIGURE 6:**
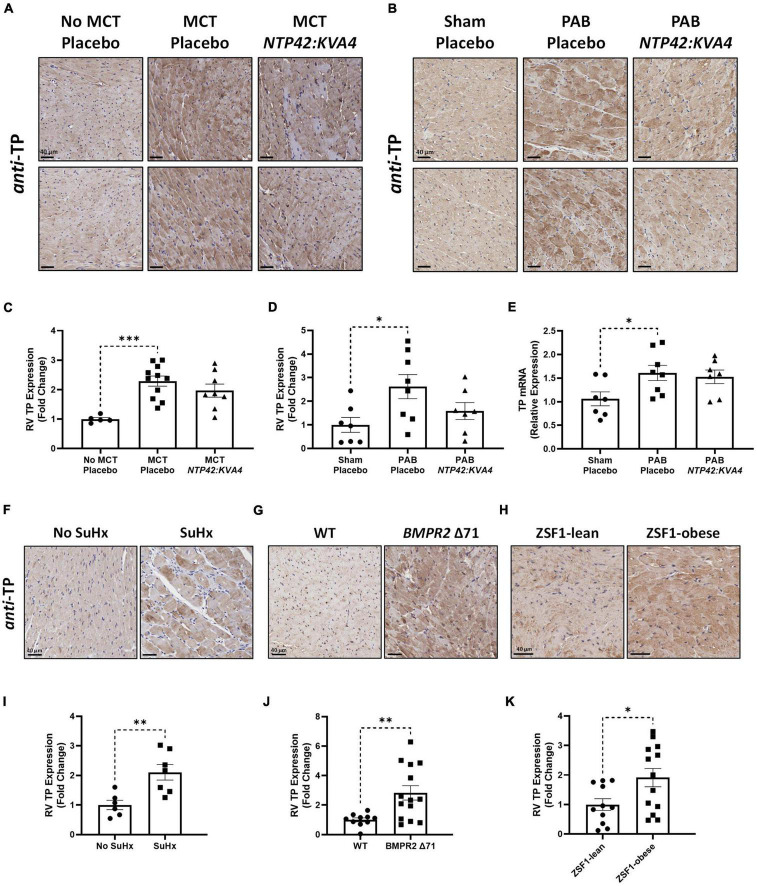
RV expression of the TP in preclinical models of PAH and RV dysfunction. **(A,B)** Show two representative photomicrographs, selected from a random animal from each treatment group, of *anti*-TP-stained RV tissue from the: **(A)** MCT-PAH model, and **(B)** PAB model, where images were captured at 400 × magnification (scale bars represent 40 μm). **(C)** Shows relative immunohistochemical (IHC) expression of the TP in the MCT-PAH model in RV tissue from animals of the “No MCT—Placebo,” “MCT—Placebo,” and *NTP42:KVA4* groups [*n* = 5, 11, and 8, respectively]. **(D–E)** Show: **(D)** relative TP IHC expression in the PAB model in RV tissue from animals of the “Sham—Placebo,” “PAB—Placebo,” and *NTP42:KVA4* groups [*n* = 7, 8, and 7, respectively], and **(E)** RV TP mRNA expression [*n* = 7, 8, 7]. **(F–H)** Show representative photomicrographs, selected from a random animal from each treatment group, of *anti*-TP-stained RV tissue from: **(F)** Control groups of a previously described SuHx-PAH model (28), namely “No SuHx” and “SuHx,” where normal control rats (“No SuHx”) and rats treated with SuHx (“SuHx”) were treated PO BID with vehicle upon removal from hypoxia and continuing in normoxia until Day 49 (i.e., 4 weeks duration); **(G)** *BMPR2* Δ 71 rats or their WT counterparts, and **(H)** ZSF1-obese rats or their lean counterparts (ZSF1-lean). **(I–K)** show relative expression levels of the TP in RV tissue from: **(I)** “No SuHx” and “SuHx” animals [*n* = 6 and 7, respectively]; **(J)** WT or *BMPR2* Δ71 animals [*n* = 14 and 11, respectively], and **(K)** ZSF1-lean or ZSF1-obese animals [*n* = 13 and 11, respectively]. Data presented are the mean ± SEM. *P < 0.05, **P < 0.01, ***P < 0.001 vs. the respective disease control group in each case according to one-way ANOVA with Holm-Šídák correction **(A–C)** or unpaired Student’s *t*-tests **(I–K)**.

While low levels of TP expression were noted in the RV myocardium in No MCT and Sham animals, increased TP expression occurred in all diseased groups (Figures 6A–D). In addition, genomic analysis confirmed elevated TP expression levels in the PAB model (Figure 6E). Administration of *NTP42:KVA4* in both MCT-PAH and PAB models led to a non-significant trend toward reduction in TP expression (*P* = 0.2091 and *P* = 0.0920, respectively; Figures 6A–D). As no significant effects on TP expression were observed following treatment with PAH SOCs in either model (Supplementary Tables 4, 5), a potential effect on TP expression following specific receptor engagement with *NTP42:KVA4* is notable and may indicate a mechanism whereby TP antagonism may lead to beneficial RV effects.

Further evidence for increased TP expression in the RV in PAH and, potentially in other cardiac dysfunctions, was found in RV specimens from both the Sugen5416/Hypoxia (SuHx)-induced PAH model (Figures 6F,I), and notably from rat strains harboring a mutation in the BMPR2 gene, the primary genetic cause of heritable PAH in humans (Figures 6G,J). Furthermore, TP expression was also increased in RVs from obese ZSF1 rats, a recognized model of heart failure with preserved ejection fraction (HFpEF) which also manifests RV dysfunction (Figures 6H,K).

**FIGURE 7:**
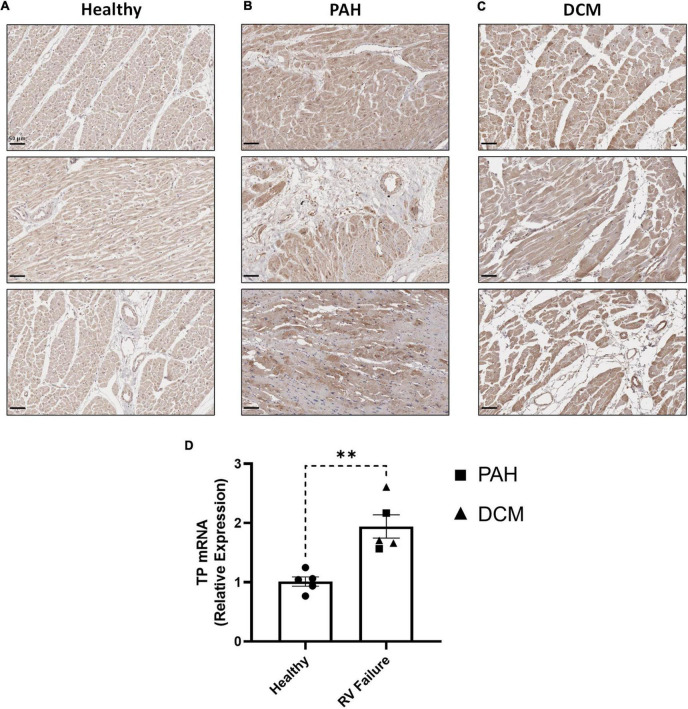
RV expression of the TP in human PAH and other RV pathologies. **(A–C)** show three representative photomicrographs of *anti*-TP-stained human RV tissue obtained from: **(A)** Healthy donors; **(B)** PAH patients, and **(C)** DCM patients, where all images were captured at 150 × magnification (scale bars represent 50 μm). **(D)** Shows relative RV TP mRNA expression levels in Healthy (*n* = 5) or RV Failure patients (*n* = 5, data for PAH and DCM combined). Data presented are the mean ± SEM. **P < 0.01 according to unpaired Student’s *t*-test.

In clinical specimens from healthy human donors (Figure 7A), TP expression was observed at a low level throughout the myocardium, with stronger expression evident in perinuclear regions, consistent with previous reports (19). In diseased tissue, TP expression was augmented in the enlarged cardiomyocytes of RV samples from PAH patients (Figure 7B), with increased expression also observed in RV samples from DCM (Figure 7C), a primary cardiomyopathy which results in ventricular dilation and functional impairment. Quantitative analysis confirmed these elevated TP expression levels in PAH & DCM cases, relative to healthy donors (Figure 7D). Notably, while increased TP expression occurred in RVs in experimental models and in clinical PAH samples, no significant changes were observed in the matching LV tissues (Supplementary Figure 4).

## Discussion

Right heart function is widely viewed as the most important determinant of clinical outcome in PAH and indeed in various other forms of pulmonary hypertension (PH) (45). Insufficient or aberrant RV adaptation, the development of RV dysfunction, and the progression to right heart failure in PAH involves complex pathological mechanisms and a precise understanding of the causes underlying these mechanisms remains to be fully elucidated (46). Unfortunately, no currently available PAH SOC therapy directly targets right heart adaptation and function.

While PAH SOCs demonstrate robust pulmonary vasodilatory enhancing effects and lead to meaningful reductions in PVR, they show limited evidence of directly targeting right heart adaptation and function. In PAH, there is an unmet need to not only alleviate pulmonary pathology and PVR, but to also directly target mechanisms underlying RV dysfunction to enhance patient quality-of-life and improve survival. Current PAH SOCs have variable effects on RV function in both experimental and clinical settings (47). In preclinical models, the prostacyclin analog Iloprost improved RV contractility, and a recent trial in PAH patients demonstrated that Iloprost increases contractility and RV-PA coupling (48). However, large-scale clinical trials of Epoprostenol in heart failure patients demonstrated an association with increased mortality (49, 50). In preclinical models, endothelin receptor blockade worsens cardiomyocyte contractility (51). Clinical trials of endothelin receptor antagonists in patients with heart failure have never fully reported, making it impossible to assess efficacy or, at worst, suggestive of unfavorable effects (52–54). In PAH patients, while acute treatment with the phosphodiesterase (PDE)5 inhibitor Sildenafil improved RV diastolic function (55), recent trials in non-PAH heart failure patients with Sildenafil or the soluble guanylate cyclase (sGC) stimulator Riociguat failed to meet their primary clinical endpoints (56, 57).

Early studies targeting the TXA_2_/TP pathway in preclinical PAH demonstrated conflicting results (58, 59). However, through recent evaluations in both the MCT- and SuHx-induced PAH models, we have demonstrated that the TP antagonist *NTP42* attenuates multiple features of experimental PAH (27, 28). In this study, the efficacy of the *NTP42:KVA4*, a novel oral formulation of *NTP42* specifically developed for clinical use and recently validated in a Phase I clinical trial (NCT04919863) in alleviating pulmonary pathologies in the MCT-PAH model was confirmed to be in line with previous findings using the *NTP42* API. Moreover, this MCT-PAH study demonstrated that *NTP42:KVA4* attenuates RV hypertrophy and promotes a more beneficial pattern of RV adaptation. Thereafter, we aimed to investigate the potential for *NTP42:KVA4* to directly target the compromised RV using the PAB model of right heart pressure overload. With an absence of confounding pulmonary pathologies, the PAB model allows for a direct assessment of therapeutic intervention on RV structure and function (60). In this PAB model, *NTP42:KVA4* reduced RV dilation as observed from ECHO and PV loop indices and also alleviated RA enlargement. While not affecting gross RV hypertrophy *per se* as evidenced by unchanged wall thickness and Fulton’s Index, *NTP42:KVA4* treatment also resulted in a more adaptive pattern of hypertrophy, with significantly decreased cardiomyocyte size and a significant increase in capillary density in RV tissue. In individually isolated cardiomyocytes, the PAB-induced increase in passive tension development, a key indicator of cell stiffness, was attenuated by *NTP42:KVA4* being indistinguishable from that of healthy control animals, and decreased degradation of the Ca^2+^-handling proteins NCX1 and SERCA2a. Moreover, due to the overall improved RV geometry, decreased RV dilation, and attenuated profiles of intrinsic diastolic and systolic cardiomyocyte tension development, treatment with *NTP42:KVA4* resulted in significantly improved RV function in PAB animals. Most notably, *NTP42:KVA4* markedly improved RV EF, near normalizing this key parameter relative to control Sham animals.

Together, the findings from these two independent preclinical models demonstrate that *NTP42:KVA4* not only alleviates pulmonary pathologies akin to those observed in clinical PAH, but also may act as a direct cardioprotective agent in settings of right heart pressure overload. Throughout these preclinical models, the efficacy seen with *NTP42:KVA4* was similar or indeed greater than the PAH SOCs used herein (Supplementary Tables 4, 5). Furthermore, in translating the preclinical efficacy findings generated in these rodent PAH models to that predicted to clinically occur in man, it is important to also note that the API *NTP42* was rationally designed and selected using the human and not the rodent TP drug target (61, 62). Thus, due to key evolutionary differences in the TP in primates vs. lower species (63), *NTP42* is a highly potent antagonist of both TPα and TPβ isoforms of the human TP (61, 62), inhibiting TXA_2_ mimetic U46619-induced calcium mobilization in cell lines stably over-expressing the human TP, and U46619-induced aggregation of human platelets, with IC_50_ values of 8.86 and 10.6 nM, respectively (27). However, in similar studies in the rat, *NTP42* is substantially less potent inhibiting U46619-induced calcium mobilization by the rat TP and aggregation of rat platelets *ex vivo* with IC_50_ values of 1.91 and 3.2 μM, respectively (Supplementary Figure 5). Thus, based on its relative IC_50_ for the TP in rats and humans, *NTP42* will be substantially more efficacious (250–300-fold) in man than in rat. Consistent with this proposition, in the recent Phase I clinical trial of the IMP *NTP42:KVA4* in healthy subjects (NCT04919863), the IC_50_ of *NTP42* for inhibition of U46619-induced aggregation of human platelets *ex vivo* was confirmed to be 9.9 nM. Extending this translation of preclinical to predicted clinical data for the PAH SOCs, based on their relative IC_50_ in rats and humans, both Sildenafil (IC_50_ in rat and man, approx. 3 nM) (64, 65) and Macitentan (IC_50_ in rat and man, 1 nM) (66, 67) are expected to be equally efficacious in both species. In contrast to this, based on its EC_50_ of 170 and 4 nM in rat and man (67, 68), the prostacyclin receptor agonist Selexipag is predicted to be 42.5-fold more efficacious in man than in rat and, therefore, is likely to elicit a better outcome clinically than observed in this or other preclinical studies. Indeed, this may account for the poor efficacy observed for Selexipag on key parameters such as mPAP, RVSP and Fulton’s Index (Supplementary Table 4). With regard to Riociguat, based on its relative potency (EC_50_ in rat and man, 30 and 80 nM, respectively) (69, 70), it is predicted to be 2.6-fold more efficacious in rat than in man, likely generating a poorer clinical outcome than it does in the preclinical studies carried out in the rat. In addition to such key species-dependent differences in target specificity, it was also notable there were fewer systemic effects apparent for *NTP42:KVA4* relative to the PAH SOCs. Specifically, while Selexipag and Riociguat led to changes in HR and mAP, respectively, and treatment with all the PAH SOCs tested led to increased liver weight indices, similar indicators of potential off-target or toxicological effects were not observed with *NTP42:KVA4* (Supplementary Tables 4, 5). In addition, while demonstrating effects in the compromised RV, PV loop analysis showed that *NTP42:KVA4* did not affect LV parameters (data not shown). As an important regulatory-compliant safety parameter required by both the European and US EMA and FDA agencies before proceeding to FIH Phase I clinical trials, the *in vivo* effect of *NTP42:KVA4* on the cardiovascular system was also investigated in conscious telemetered dogs, where no inotropic or chronotropic effects were observed at doses up to 450 mg/kg *NTP42:KVA4* PO (Supplementary Figure 6).

In previous studies investigating the role of the TXA_2_/TP pathway on RV dysfunction, TP antagonism was protective against mild RV pressure overload in a mouse PAB model, where pressures and cardiac output were improved (19). TP antagonism also attenuated PAB-induced increases in end-diastolic calcium levels and improved cardiac repolarization and reduced ECG abnormalities through restoration of the gap junction protein connexin 43 (71–73). Targeting of TXA_2_/TP pathway has been previously investigated in PAH clinical therapy. Terbogrel, a dual TP antagonist and TXA_2_ synthase (TXAS) inhibitor was evaluated in a Phase II clinical trial, but this study was prematurely terminated during enrolment due to the development of acute leg pain in trial participants (74). As subsequently reported, this leg pain occurred due to Terbogrel’s inhibition of TXAS which, while blocking TXA_2_ generation, resulted in a shift toward synthesis of prostacyclin, a potent pain inducer. In contrast to Terbogrel, *NTP42* is a highly selective TP antagonist, which does not inhibit TXAS and therefore, as also confirmed in the recent Phase I clinical trial even at high doses, will not induce leg pain (27). In addition, the specificity of *NTP42* for the TP has been previously reported, with no agonist or antagonist activity at the 7 other prostanoid receptors, namely the prostaglandin (PG) D_2_ (DP_1_), PGE_2_ (EP_1_, EP_2_, EP_3_, EP_4_), PGF_2α_ (FP) and PGI_2_/prostacyclin (IP) receptors, with no agonist activity at the TP itself (27).

**FIGURE 8:**
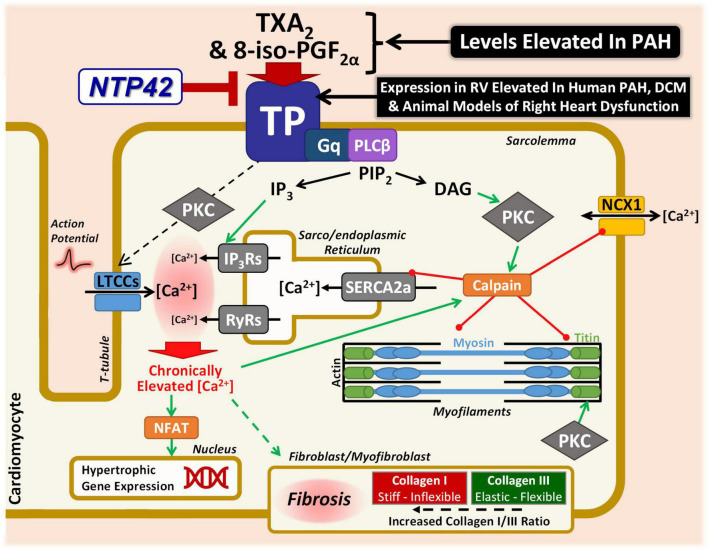
Specific antagonism of TP signaling may alleviate key mechanisms involved in cardiac dysfunction in PAH and other cardiac conditions. Schematic representation focusing on the potential mechanisms through which the TXA_2_/TP signaling axis may lead to cardiac dysfunction in PAH and other cardiac conditions. Signaling through the Gq-coupled TP leads to PLCβ activation, liberating IP_3_ and DAG from PIP_2_. IP_3_ release mobilizes Ca^2+^ from the sarco/endoplasmic reticulum via IP_3_R activation. IP_3_-mobilized Ca^2+^, along with extracellular Ca^2+^ influx via LTCCs, triggers a larger Ca^2+^ release via RyRs. LTCC-mediated Ca^2+^ influx may also occur via a TP-mediated PKC mechanism. Chronically elevated Ca^2+^ leads to hypertrophic signaling via NFAT. Ca^2+^ overload also activates the protease calpain, which degrades myofibrillar proteins including myosin and titin, as well as Ca^2+^-handling proteins including NCX1 and SERCA2a. This study has demonstrated decreased degradation of both NCX1 and SERCA2a following *NTP42:KVA4* treatment. Furthermore, PKC-mediated phosphorylation of titin leads to increased stiffness, and it is hypothesized that the decreased cardiomyocyte stiffness observed herein following TP antagonism may be as a result of reduced PKC-mediated titin phosphorylation. In addition, TP antagonism may also act on the cardiac fibroblast/myofibroblast, decreasing cardiomyocyte stiffness by resulting in a decreased Collagen I/III ratio and/or net RV fibrosis levels, as demonstrated in this study. Finally, not only are levels of the TP ligands TXA_2_ and 8-iso-PGF_2α_ increased in PAH and in other cardiopulmonary conditions, but this study has demonstrated elevated expression of the TP in the myocardium in clinical PAH and dilated cardiomyopathy (DCM), as well as in multiple independent experimental models of PAH and/or cardiac dysfunction. Both TXA_2_ and 8-iso-PGF_2α_ would be predicted to further compound the contribution of the TXA_2_/TP pathway to detrimental effects within the diseased myocardium.

As depicted in the model in Figure 8, there are many putative mechanisms by which signaling via the TXA_2_/TP pathway may elicit detrimental effects within the myocardium, where the findings from this study provide important mechanistic insights into this and how *NTP42* alleviates this dysfunction. Consistent with a large body of data, including from this laboratory (63), signaling through the TP, TXA_2_ induces profound increases in intracellular calcium (Ca^2+^) in many cell types. Specifically, in cardiomyocytes, basal and peak Ca^2+^ concentrations as well as width of Ca^2+^ transients are increased following treatment with the TXA_2_ mimetic U46619, and prolonged stimulation results in irregular Ca^2+^ oscillations and a marked increase in cytosolic-free Ca^2+^ concentrations (20). Direct injections of U46619 also induce ventricular arrhythmia in rabbits, where this effect occurs through a mechanism independent of reductions in coronary blood flow or activation of the autonomic nervous system (22). As a G protein-coupled receptor, the TP primarily couples to Gq, resulting in phospholipase (PL)Cβ activation and liberation of inositol trisphosphate (IP_3_) and diacylglycerol (DAG) from phosphatidylinositol 4,5-bisphosphate (PIP_2_) cellular stores (63). IP_3_ release leads to rapid mobilization of intracellular Ca^2+^ from the sarco/endoplasmic reticulum (SR) via activation of ligand-gated IP_3_ receptors (IP_3_Rs). In this regard, treatment of isolated cardiomyocytes with U46619 increased intracellular Ca^2+^ in a dose-dependent manner, where these increases were blocked by the TP antagonist SQ29548 or inhibitors of the IP_3_ pathway (23). IP_3_-mobilized Ca^2+^, along with the depolarization phase of the action potential and extracellular Ca^2+^ influx via L-type Ca^2+^ channels (LTCCs), triggers a larger Ca^2+^ release from the SR via ryanodine receptors (RyRs), leading to cardiac contraction. A further mechanism through which the TP may lead to increased intracellular Ca^2+^ is following protein kinase (PK) C activation due to second-messenger DAG liberation from PIP_2_. It has been shown by multiple groups that TP stimulation in vascular smooth muscle cells leads to dysregulation of resting membrane potential and subsequent activation of LTCCs (75–78). Mechanistically, Cogolludo et al. demonstrated that U46619 treatment of pulmonary artery smooth muscle cells directly inhibits voltage-gated K^+^ channels via a PKCζ-mediated mechanism, leading to subsequent depolarization, LTCC-mediated increase in intracellular Ca^2+^, and vasoconstriction (76). Chronically elevated or dysregulated intracellular Ca^2+^ cycling plays a central role in hypertrophic signaling in cardiomyocytes (79). In particular, the distinct Ca^2+^ duty cycle produced following IP_3_-mediated Ca^2+^ release has been shown to activate pro-hypertrophic pathways, including those involving nuclear factor of activated T cells (NFAT) transcriptional mechanisms (80–82). Notably in the context of TP-mediated signaling, Ca^2+^ overload, aberrant Ca^2+^ cycling, or PKC-mediated phosphorylation, activates the cysteine protease calpain (83, 84). In turn, activated calpain proteolytically degrades myofibrillar proteins including myosin and titin, as well as Ca^2+^-handling proteins including NCX1 and SERCA2a (85–87). Herein, this study shows decreased degradation of both NCX1 and SERCA2a following *NTP42:KVA4* treatment in the PAB model. We propose that this is one possible mechanism where *NTP42* antagonism of TP signaling may be involved in preventing progression to cardiac dysfunction and heart failure. Besides this, there are other mechanisms which may drive cardiac dysfunction and account for the observed benefits of *NTP42*, including potentially involving free-radical mechanisms and/or contractile machinery modifications (35, 36). Levels of 8-iso-PGF_2α_, a non- enzymatic-, free-radical- derived product of arachidonic acid, are increased in line with heart failure severity and associated with increased ventricular dilation (88). Notably, the TP also mediates the actions of 8-iso-PGF_2α_, where uniquely TP antagonism is predicted to have the additional benefit of blocking this important pathological mediator of oxidative injury. Furthermore, in the context of the myofibril machinery, as a key determinant of myocardial passive stiffness, the distensibility of titin is heavily regulated by phosphorylation (89). In contrast to PKA/PKG-mediated effects, PKC phosphorylation of titin is widely known to increase cardiomyocyte stiffness (90–92). Mechanistically, as a Gq-coupled receptor directly linked to PKC activation (63), a plausible working hypothesis of how TP antagonism by *NTP42* may lead to decreased stiffness is by reducing PKC-mediated titin phosphorylation. While beyond the scope of this study, further mechanistic investigations are warranted to explore these proposed TXA_2_/TP-mediated mechanisms.

Several limitations with the current study are acknowledged. While anesthesia may lead to depressed cardiac function and systemic hemodynamic effects, the anesthetics isoflurane or sevoflurane used in our studies are known to have only mild effects on cardiac function in rodents (46, 93). Furthermore, as anesthetic regimens were used identically in all animal groups, we would not contend that this affected the study findings. Regarding the pathologies seen in the MCT-PAH and PAB models, it is accepted that these develop in a short time frame and that these preclinical studies may not completely recapitulate the changes that develop progressively over many years in the human condition. However, it is important to acknowledge that our data shows that increased expression of the TP occurred in RVs of several independent and relevant preclinical models, including in the MCT- and SuHx-PAH models, in the PAB model of RV overload, in the ZSF-1 model of heart failure and spontaneously in the *BMPR* Δ71 rodents without intervention. Importantly, increased expression of the TP was also found in clinical RV specimens from subjects with PAH as well as from DCM subjects. While historically most clinical attention in the study of DCM has been LV function and morphology, recent advances in cardiac imaging show that RV involvement is common in DCM, and the presence of RV dysfunction is in fact a major negative prognostic determinant in DCM morbidity and mortality (94). The observations reported herein for the potential involvement of the TP and the TXA_2_ pathway in DCM pathogenesis or progression warrant further investigation in relevant preclinical models. The studies reported herein are in line with the current recommendations that, where possible, the effects of an intervention be tested in multiple animal models (27–29). With regard to the choice of preclinical models, and the specific timing of intervention used herein, treatment with *NTP42:KVA4* or PAH SOC was commenced in as delayed a schedule as possible while still permitting sufficient animal survival for analyses through complementary modalities, including invasive assessments of pressure-volume relationships. While the findings from the early interventional MCT-PAH approach reported herein add substantially to previous reports from a preventative MCT-PAH model (27) it is acknowledged that the therapeutic effects of *NTP42:KVA4* could be further investigated using conditions viewed as more reminiscent of a reversal approach, such as by further delaying treatment in an MCT-PAH or PAB model. Finally, regarding the findings from the PAB model, while *NTP42:KVA4* treatment resulted in normalization of isolated cardiomyocyte passive tension, statistical significance for the functional consequence of this improvement in diastolic stiffness, such as in Eed, was not achieved (*P* = 0.091, Figure 3J). While trending toward benefit, a possible explanation for this disparity is that while Eed measurement corresponds with the stiffness of the heart *in vivo*, tension measurements are taken *ex vivo* from individual isolated cardiomyocytes. While this isolation procedure preserves the structural and functional properties of the myofibrillar apparatus, these cells present with sarcolemmal damage and loss of intracellular organelles (95). These isolated cardiomyocytes also lack supporting extracellular matrix, fibrotic deposition, or cell-cell interactions that may contribute to diastolic stiffness *in vivo* (95). In addition, while assessments of NCX-1 expression showed unchanged levels of intact functional protein following PAB, the finding of increased degraded protein likely indicates that net expression of NCX-1 may be increased upon PAB, presumably as a compensatory or adaptative mechanism to cope with elevated Ca^2+^ levels and aberrant Ca^2+^ cycling in the compromised cardiomyocyte. Increased NCX-1 degradation in this setting would hence be predicted to have a negative effect on this adaptive response. However, and as demonstrated in this study, treatment with *NTP42:KVA4* led to significantly decreased levels of NCX-1 degradation and this benefit on NCX-1 turnover is hypothesized to be a component of the mechanism of TP antagonism in this model.

In conclusion, these preclinical studies provide evidence that, through antagonism of TP signaling, *NTP42*, administered orally as the clinical formulation *NTP42:KVA4*, may attenuate PAH pathophysiology by not only alleviating pulmonary pathologies but also by reducing RV remodeling and promoting beneficial hypertrophy, resulting in improved cardiac function. These findings in experimental models point to a cardioprotective effect for *NTP42:KVA4* as a component of its therapeutic potential not only in PAH, but possibly in other RV dysfunctions. Finally, expanding on the growing evidence for the role for the TP in PAH and the potential for antagonism of the TP as a therapeutic strategy in its clinical management, such as with *NTP42:KVA4*, this current study also demonstrated elevated expression of the TP in RV tissue from human PAH and other cardiomyopathy patients, validating the potential of this largely ignored target ripe for pharmaceutical intervention.

## Data availability statement

The raw data supporting the conclusions of this article will be made available by the authors, without undue reservation.

## Ethics statement

Protocols for the collection of human biomaterials used in this study were reviewed and approved by the Institute of Cardiometabolism and Nutrition BioCollection (Paris, France). All patients/participants, or their surrogates, provided written informed consent for the use of these biomaterials for research purposes. Animal studies were reviewed and approved by the Institutional Animal Care and Use Committee of IPS Therapeutique (Sherbrooke, QC, Canada), or by the Ethical Committee of the University of Porto (Porto, Portugal), certified by the Portuguese National Authority for Animal Health.

## Author contributions

EM, FR, RA, ED, LB, VS, HR, GC, JG, AL-V, J-BM, CB-S, CL, LH, DM, MH, AV, FP, PM-F, and BK: substantial contributions to the conception or experimental design of the work or the acquisition, analysis, and interpretation of data for the work. EM, LH, DM, MH, AV, FP, PM-F, and BK: drafting the manuscript or revising it critically for important intellectual content. All authors have read and approved the final manuscript.
