# Supplementary material for: The thromboxane receptor antagonist NTP42 promotes beneficial adaptation and preserves cardiac function in experimental models of right heart overload
Source: Front Cardiovasc Med. 2022 Dec 14;9:1063967. doi: 10.3389/fcvm.2022.1063967 (PMC9794752; doi:10.3389/fcvm.2022.1063967)
Supplement: Supplementary file 1 [file Presentation_1.pdf]

SUPPLEMENTAL MATERIAL

SUPPLEMENTAL METHODS

NTP42:KVA4 formulation

To enhance oral bioavailability and for use as an investigational medicinal product (IMP), the active pharmaceutical ingredient (API) *NTP42* was blended in a 1:4 w/w ratio with the pharmaceutical excipient Kollidon® VA 64, where this IMP is hereafter referred to as *NTP42:KVA4*. Thus, 1 part of *NTP42:KVA4* is equivalent to 0.2 parts *NTP42*, and to calculate active *NTP42* dosages it is necessary divide *NTP42:KVA4* dose levels by 5.

Monocrotaline-induced model of preclinical pulmonary arterial hypertension

Monocrotaline (MCT)-induced PAH animal studies were performed at the facilities of IPS Therapeutique (Sherbrooke, Quebec, Canada). The Institutional Animal Care and Use Committee of IPS Therapeutique approved the studies in strict accordance with the guidelines of the Canadian Council on Animal Care and the US NIH Guide for the Care and Use of Laboratory Animals and the International Association for Assessment and Accreditation of Laboratory Animal Care. Male Sprague-Dawley rats (Charles River Laboratories) aged between 7 and 9 weeks and weighing 309 – 417 g at the time of their enrolment in the study were randomized according to their body weight into 7 groups (Groups 1 – 7; see **Table S1A**). Animals in Groups 2 to 7 received a single dosage of MCT (AdooQ Bioscience, Irvine, CA) by subcutaneous injection at 60 mg/kg dosage (2 mL; 30 mg/mL stock, in dimethyl sulfoxide, DMSO) on the morning of Day 0. Animals in Group 1 received one subcutaneous injection of the MCT vehicle (DMSO; 2 mL/kg) on the morning of Day 0. Drug treatments were initiated on Day 7 and continued until Day 28. During this period, animals were treated by oral gavage (PO) twice-daily (BID) with the IMP *NTP42:KVA4* (1 mg/kg, equivalent to 0.2 mg/kg *NTP42* API, Group 3), Sildenafil (50 mg/kg, Group 4), Macitentan (30 mg/kg, Group 5), Selexipag (1 mg/kg, Group 6), Riociguat (5 mg/kg, Group 7) or, as negative control, with placebo (30 mg/kg Kollidon® VA 64; Groups 1 and 2). MCT, Sildenafil, Macitentan, Selexipag, and Riociguat were obtained from AdooQ Bioscience (Irvine, Ca, USA). In all cases, treatments or placebo were delivered as a suspension in 0.5 % (w/v) hydroxypropyl methylcellulose (HPMC) E3. During the treatment period, rats were given food and water *ad libitum*. The animals were pair-housed for the duration of the study. All animal care and vivarium maintenance were recorded, with documents kept at the test facility. In addition, clinical observations or cage-side parameters were also recorded throughout the study including food and water intake, breathing activity levels, clinical signs of distress, general well-being, and changes in body weight.

On the final day of the study (Day 29), animals were anesthetized with a mixture of 2 to 2.5 % isoflurane in oxygen. Hemodynamic and functional parameters [mean pulmonary arterial pressure (mPAP), right ventricular (RV) systolic pressure (RVSP), mean systemic arterial pressure (mAP), and heart rate (HR)] were recorded continuously for 30 seconds or until loss of pulmonary arterial (PA) pressure signal, whichever came first. The rats were then exsanguinated, and the lungs and heart were removed *en bloc* from the thoracic cavity and were weighed. The right lobes were clamped, and the left lobes were flushed with 0.9% NaCl and then perfused with 10% NBF (10% neutral buffered formalin). The heart and right lobes were weighed to estimate the weight of the left lobe. The cardiac tissues were excised to measure the wet weights of the RV and left ventricle (LV) including the septum as part of the Fulton's Index, and the RV was transferred to 10% NBF. The right tibia length (TL) was also measured, and the liver weight was also recorded.

Pulmonary arterial banding model of right heart overload

Pulmonary arterial banding (PAB) animal studies were performed at the facilities of the Faculty of Medicine, University of Porto (Porto, Portugal). All animal experiments in this study were in accordance with the recommendations of the US NIH Guide for the Care and Use of Laboratory Animals and were approved by the ethical committee of the University of Porto and certified by the Portuguese National Authority for Animal Health (Approval Number: 0421/000/000/2013). Male Sprague-Dawley rats (Charles River Laboratories) aged between 7 and 9 weeks and weighing 190 – 251 g at the time of their enrolment in the study were randomized according to their body weight into 4 groups (Groups 1 – 4; see **Table S1B**). Animals in Groups 2 to 4 were pre-treated with buprenorphine for analgesia (0.1 mg/kg) and were anesthetized with 8% sevoflurane inhalation. Following intubation, animals were connected to a rodent ventilator (VentElite, Harvard Apparatus), with tidal volume and respiratory rate adjusted to animal weight, and a heating pad with temperature control. Anesthesia was kept at 2.5 – 3.0 % sevoflurane mixed with 100% O_2. The animals were placed in the right lateral decubitus position, and after depilation and disinfection of the left thorax, an incision was made at the axillary level. The pectoral muscles were dissected and retracted, and an incision was made in the third intercostal space, exposing the PA, left atrium, and aorta. After separation of the PA, a 5-0 polypropylene suture was passed around and tied with two double knots against an 18G needle, resulting in a constriction to a fixed diameter. After thorax closure, the pectoral muscles were moved back into place, and the skin was closed with 6-0 or 7-0 polypropylene suture, followed by infiltration with 1% lidocaine. Animals from Group 1 ('Sham – Placebo') underwent a similar procedure, except where the suture around the PA was loosely tied.

Immediately following PAB surgery, rats were repositioned in the left lateral decubitus position. An ultrasound system (Acuson Sequoia 512, Siemens) equipped with a 5-7-MHz probe (model 3V2c) was used for echocardiogram (ECHO) assessment of the pressure gradient across the PA constriction. Only animals where a peak PA pressure gradient ≥ 20 mmHg after surgery was observed were included in the final analysis set. After the ECHO assessment, the chest was cleaned, and povidone-iodine solution was applied to the incision site. The animals were allowed to recover in a clean cage, under infrared light, until a full recovery was observed. Analgesia was continued for 48 hours or was continued for longer if requested by the veterinary staff.

Thereafter, drug treatments were initiated on Day 2 post-PAB surgery and continued until Day 27. During this period, animals were treated PO BID with the IMP *NTP42:KVA4* (1 mg/kg, equivalent to 0.2 mg/kg *NTP42* API, Group 3), Riociguat (5 mg/kg, Group 4) or, as negative control, with placebo (30 mg/kg Kollidon® VA 64; Groups 1 and 2). Riociguat was obtained from AdooQ Bioscience (Irvine, Ca, USA). During the treatment period, rats were given food and water *ad libitum*. The animals were pair-housed for the duration of the study. All animal care and vivarium maintenance were recorded, with documents kept at the test facility. In addition, clinical observations or cage-side parameters were also recorded throughout the study including food and water intake, breathing activity levels, clinical signs of distress, general well-being, and changes in body weight.

On the final day of the study (Day 28), ECHO assessments, as detailed above, were carried out on all animals prior to terminal hemodynamic surgery. The echocardiographic parameters collected included RV end-diastolic dimension (RVEDD; apical four-chamber [A4C] view), RV end-diastolic area (RVEDA; parasternal short-axis [PSAX] view), RV free wall thickness at diastole (RVFWT; M-mode viewing), and maximum right atrial (RA) area (RAA; A4C view). Following ECHO assessments, the animal was returned to the right lateral decubitus

position for pressure-volume catheterization. The common femoral vein was catheterized for fluid administration, and a left thoracotomy was performed; the pericardium and pleura were carefully dissected, and the phrenic nerve was severed. A 3-0 surgical silk was passed around the inferior vena cava for transient occlusion during the protocol, and pressure-volume (PV) catheters were inserted through the apex of the RV and LV (models SPR-869 and SPR-847, respectively, Millar Instruments) and positioned along the long axis. A flow probe (model MA2.5PSB, 2.5 mm, Precision S-Series, Transonic Systems) was implanted around the ascending aorta and connected to an ultrasonic transit time-volume flowmeter (model TS420, transit-time perivascular flowmeter, Transonic Systems). The experimental setup was allowed to stabilize for 15 min and any blood loss during the procedure was replaced with a saline bolus. PV signals were continuously acquired (model MVPS 300, Millar instruments), digitally recorded at a sampling rate of 1 kHz (model ML880 PowerLab 16/30, ADInstruments), and analyzed offline (LabChart 8 Pro, ADInstruments). Parallel conductance for the volume catheter was computed after an average of three bolus injections of 50 µL of hypertonic saline (10% NaCl). Calibration for factor alpha (field inhomogeneity) was determined through the cardiac output (CO) measured by the aortic flow probe and the ultrasonic transit time-volume flowmeter. Parameters collected from PV catheterization included HR, mAP, RV end-systolic pressure (ESP), RV end-diastolic pressure (EDP), RV end-diastolic volume (EDV), RV end-systolic volume (ESV), CO and RV ejection fraction (EF). The PV relationships end-systolic elastance (E_{es}) and end-diastolic elastance (E_{ed}) were calculated from the ESP- and EDP-volume slopes, respectively.

Once the protocol was completed, the rats were exsanguinated, and the lungs and heart were removed *en bloc* from the thoracic cavity and were weighed. The cardiac tissues were excised to measure the wet weights of the RV and LV including the septum as part of the Fulton's Index and were each transferred to 10% NBF. During organ harvesting, a sample of RV free wall from up to 6 randomly-selected animals per treatment group was collected for isolated cardiomyocyte analysis, as detailed below, and was snap frozen in liquid nitrogen and stored at -80 ºC until use. In addition, RV samples were dissected from the RV free wall at the end where it connects with the interventricular septum/LV and equilibrated in RNAlater® solution (ThermoFisher), prior to storage at −80 °C until RNA isolation was performed. In addition, the RA wall weight and the liver weight was also recorded.

Pulmonary histology and analysis

Formalin-fixed paraffin-embedded (FFPE) tissue blocks from the middle region of the left lung from MCT-PAH study animals were sectioned at 4 µm. The sections were baked onto slides at 50–56 °C for 60 min. Prior to staining, sections were dewaxed in two changes of xylene (2 × 10 min incubations) and rehydrated through a series of decreasing alcohol solutions (100%, 3 min × 2; 95%, 1 min; 80%, 1 min) before being washed in double-distilled water (ddH$_2$O).

For analysis of pulmonary vascular remodeling, lung sections were stained with hematoxylin and eosin (H&E) or were subject to immunohistochemical (IHC) staining for α-smooth muscle actin (SMA), where these protocols have been previously described.(1) For analysis of perivascular pulmonary fibrosis, lung sections were stained with Masson's trichrome connective tissue staining kit, according to the manufacturer's instructions (ab150686; Abcam). Trichrome-stained tissue sections were scanned at 40X magnification using an Aperio AT2 and viewed using QuPath Quantitative Pathology & Bioimage Analysis software platform.(2) To quantify the prevalence of perivascular pulmonary fibrosis, all small pulmonary arterioles (10-50 µm in diameter) across 3 randomly selected fields/section (3 mm^2 total tissue area) were counted and classified as either non-fibrotic (no apparent collagen deposition surrounding

arteriole), partially fibrotic (crescent of collagen deposition visible) or fully fibrotic (complete adventitial layer of collagen deposition) by visual inspection. All analyses were carried out in a blinded fashion with overlap by three independent observers. For quantitation of pulmonary macrophages, sections were stained with *anti*-CD (cluster of differentiation) 68 antibody (Abcam; ab31630). Antigen retrieval was first performed by boiling the slides in sodium citrate buffer, in a pressure cooker for 10 min. Endogenous peroxidase activity was blocked by incubating the slides in hydrogen peroxide (3% v/v prepared in absolute methanol) for 30 min at room temperature (RT). To block non-specific binding and endogenous biotin, the tissue sections were incubated for 30 min at RT with Blocking Buffer (5% goat serum in phosphate-buffered saline, PBS) containing Avidin D (4 drops/mL; Avidin/Biotin Blocking Kit, Vector Laboratories). Sections were then incubated with *anti*-CD68 antibody (1:1000 dilution) overnight at 4 °C in a humidified chamber. *anti*-CD68 antibody was diluted in Blocking Buffer, containing Biotin (4 drops/mL; Avidin/Biotin Blocking Kit, Vector Laboratories). Following incubation with primary antibody, sections were washed in PBS (3 × 5 min washes) and incubated for 60 min with a biotinylated *anti*-mouse immunoglobulin G (IgG) secondary antibody prepared in Blocking Buffer (1:3000). Sections were then washed in PBS (3 × 5 min washes), followed by incubation with streptavidin-conjugated horseradish peroxidase (diluted 1:1500 in Blocking Buffer) for 30 min at RT. Thereafter, the slides were incubated with the chromogen 3,3'diaminobenzidine (DAB) substrate for 4 min. Finally, tissue sections were counterstained for 1 min with Harris modified hematoxylin. Sections were then dehydrated through increasing alcohol series (80%, 2 min; 95%, 2 min; 100%, 5 min × 2) and xylene (2 × 10 min), prior to mounting in dibutylphthalate polystyrene xylene (DPX). *Anti*-CD68-stained tissue sections were scanned at 40X magnification using an Aperio AT2 and viewed using QuPath. On *anti*-CD68-stained sections, the number of $CD68^+$ cells were counted by visual inspection across 8 randomly selected fields/section (1.28 mm^2 total tissue area). All cells with identifiable staining were counted and all analyses were carried out in a blinded fashion by three independent observers.

Cardiac histology and analysis

FFPE tissue blocks from the RV from MCT-PAH study animals, or from both the RV and LV from PAB study animals, were sectioned at 4 μm and were baked, dewaxed, and rehydrated as detailed above. Cardiac sections were subject to IHC staining for CD31 or were stained with Masson's trichrome, where these protocols have been previously described.(1) *Anti*-CD31- and trichrome-stained tissue sections were scanned at 40X magnification using an Aperio AT2 and viewed using QuPath.

Morphometric analysis to evaluate cardiomyocyte cross-sectional area (CSA) along with ventricular vascularization was carried out on *anti*-CD31-stained sections, as previously described.(1) Per previous reported studies, measurement of these two parameters: (i) vascularization per unit area, and (ii) cardiomyocyte CSA, provides a direct assessment of 'metabolic supply' and 'metabolic demand', respectively, within the RV tissue and when expressed as a ratio, can be used to calculate an 'RV Adaptation Index' to discriminate between adaptive versus maladaptive hypertrophic responses.(1) The values for this index were calculated herein using: RV Adaptation Index = Ventricular Vascularization (Capillaries/mm^2) / Cardiomyocyte CSA (μm^2), where the resulting value was normalized to the mean of the No MCT or Sham control groups within each of the MCT-PAH or PAB studies, respectively. In this ratio, maladaptive hypertrophy is defined to occur where the supply (capillaries per unit area) does not keep pace with the demand (cardiomyocyte size). In this case, the RV Adaptation Index would be significantly less than 1. Conversely, where sufficient neovascularization

occurs alongside the increased RV cardiomyocyte size, the supply keeps pace with the demand, and this is termed adaptive hypertrophy. Here, in this case of adaptive hypertrophy, the value for the RV Adaptation Index would be closer to, or equal to, 1.

Trichrome-stained RV sections from MCT-PAH study animals were viewed in QuPath and 20 fields/section (20 x 0.0625 mm^2) were randomly selected. The percentage fibrotic area was determined in each field using the Fiji distribution of NIH ImageJ software(3) where images were first subjected to color deconvolution to separate the collagen (blue) staining channel, essentially as previously described.(4) Values are reported as the mean percentage fibrotic area in each section where the resulting value was normalized to the mean of the No MCT control group. All analyses were carried out in a blinded fashion with overlap by three independent observers. For PAB study animals, both trichrome-stained RV and LV sections were viewed in QuPath, and an annotation was demarcated comprising either the entire tissue area in the case of the RV, or the ventricular free wall excluding septal regions in the case of the LV, where regions containing major coronary vasculature were removed. The percentage of fibrotic area, normalized to the mean of the Sham control group, was determined in each annotated section using QuPath's automatic pixel classifier (Artificial Neural Network type; high resolution, 1.01 μm/pixel), where the classifier was manually trained to discriminate fibrotic and non-fibrotic elements on randomly selected regions within each staining run.

Isolated skinned cardiomyocytes

Force transduction experiments using isolated skinned cardiomyocytes were performed as previously described.(5, 6) In brief, RV tissue samples from PAB study animals were defrosted, mechanically disrupted, and permeabilized with 0.1% Triton X-100. Under a microscope (Model 1X51, Olympus) and using image visualization software (VSL 900B, Aurora Scientific), single cardiomyocytes were attached to a force transducer (Model 403A, Aurora Scientific) and a length controller (Model 315C-I, Aurora Scientific). Cell length was digitally adjusted through custom-designed software (Series 600A Digital Controller, Aurora Scientific), and steady-state passive tension (PT) was measured at increasing sarcomere lengths (1.8 – 2.3 μm). Total tension development was measured at a sarcomere length of 2.2 μm by varying submaximal free Ca^{2+} concentration ([Ca^{2+}], 1 – 31.6 μmol/L), and active tension (AT) was determined by subtracting PT from total tension. Calcium force response curves were fitted using nonlinear regression for each individual cardiomyocyte, and the Ca^{2+} sensitivity (half maximal effective concentration; EC_{50}) values for each cardiomyocyte were determined from the individual regression analyses, using GraphPad Prism (Version 9, GraphPad Software). In all cases, tension measurements were normalized to individual cardiomyocyte CSA.

Human cardiac tissue

Fresh frozen samples and FFPE blocks of RV and LV tissue from healthy donors, or patients with PAH or dilated cardiomyopathy (DCM), following autopsy, were obtained from the Institute of Cardiometabolism and Nutrition BioCollection (Paris, France), where patient and sample details are listed in **Table S2**. All protocols to obtain human biomaterials conformed with the recommendations of the Declaration of Helsinki, where the Institute of Cardiometabolism and Nutrition review board approved these protocols and informed written consent was obtained from patients or their surrogates for the use of biomaterials for research purposes.

RNA isolation and quantitative real-time PCR

Total RNA from RV tissue samples of PAB study animals or human cardiac tissue was isolated using the Invitrogen™ PARIS™ Kit (ThermoFisher) according to the manufacturer's instructions, after homogenization in Cell Disruption Buffer (ThermoFisher) with zirconium oxide beads (1 mm; Thistle Scientific) in a FastPrep-24 5G benchtop homogenizer (MP Biomedicals). Frozen human cardiac tissue samples were first pulverized in liquid nitrogen using a mortar and a pestle, prior to isolation of total RNA as detailed above. Following DNase treatment to remove any contaminating genomic DNA, total RNA was reverse transcribed into complementary DNA (cDNA) using Invitrogen™ SuperScript™ III Reverse Transcriptase (ThermoFisher) following the manufacturer's recommended protocol. Quantitative real-time PCR (qPCR) was performed with an Applied Biosystems™ QuantStudio™ 7 Flex PCR system using gene specific TaqMan® expression assays (See **Table S3** for individual assay IDs). In all cases, mRNA expression levels were calculated by the $2^{-\Delta\Delta CT}$ method relative to the Sham control group or Healthy donor group, where the housekeeping gene glyceraldehyde-3-phosphate dehydrogenase (GAPDH) was used for normalization throughout. In the case of *Col1a1* and *Col3a1*, the collagen I/III ratio was subsequently calculated for each RV tissue sample from the respective relative mRNA expression levels.

Ca^{2+}-handling protein expression

Lysates from RV tissue samples of PAB study animals were isolated using the Invitrogen™ PARIS™ Kit (ThermoFisher) according to the manufacturer's instructions, after homogenization in Cell Disruption Buffer (ThermoFisher) with zirconium oxide beads (1 mm; Thistle Scientific) in a FastPrep-24 5G benchtop homogenizer (MP Biomedicals). Following protein quantification (Bradford protein assay; Bio-Rad), lysates were separated by SDS-PAGE, and western blots were subjected to immunoblotting with monoclonal antibodies directed to Na^{+}/Ca^{2+} exchanger 1 (NCX1; ab177952; Abcam; 1:2000 dilution) or sarco/endoplasmic reticulum Ca^{2+}-ATPase 2 (SERCA2; ab150435; Abcam; 1:10000 dilution), as indicated in the figure legends, followed by re-probing of immunoblots for GAPDH (ab8245; Abcam; 1:5000 dilution) which served as protein loading control. Western blots were visualized using the Fusion FX chemiluminescence smart imaging system (Vilber) and normalized expression levels for NCX1 and SERCA2 (full-length protein and degraded fragment bands) were determined by semi-quantitative densitometry.

Thromboxane A_2 receptor immunohistochemistry

For investigations of thromboxane A_2 receptor (TP) expression, RV sections from MCT-PAH study animals, along with RV and LV sections from PAB study animals, as well as RV and LV sections from human cardiac tissue samples were stained with *anti*-TP antibody (HPA077366; Atlas Antibodies). RV tissues obtained from a Sugen5416/Hypoxia (SuHx)-induced PAH preclinical model were also included in this analysis, where this study has previously been described.(1) In addition, RV and LV tissues were obtained from a bone morphogenetic protein receptor 2 (BMPR2) mutant rat strain with a monoallelic deletion of 71 bp in exon 1 (*BMPR2* Δ71 rats) or their wild-type (WT) counterparts, where these animals have been previously described.(7) Furthermore, RV and LV tissues were obtained from ZSF1 rats, being first generation hybrids between the female Zucker diabetic fatty (ZDF) rat and the male spontaneously hypertensive heart failure (SHHF) rat, and where these animals have been previously described.(8) In brief, while both lean and obese ZSF1 animals are hypertensive as they inherit the hypertension gene from the male SHHF rat, the obese ZSF1 animal, inheriting

leptin receptor mutations from both female ZDF and male SHHF rats, develops heart failure with preserved ejection fraction (HFpEF) and also manifest RV hypertrophy and dysfunction.(8)

IHC procedures for staining of the TP were identical to those outlined for *anti*-CD68 IHC above, except where sections were incubated overnight with *anti*-TP primary antibody (1:100 dilution), with a biotinylated *anti*-rabbit IgG secondary antibody (1:500) for 30 min, and where a DAB incubation time of 2.5 min was used. *Anti*-TP-stained tissue sections were scanned at 40X magnification using an Aperio AT2 and viewed using QuPath. In the animal models reported herein, 20-30 random fields (each 0.0625 mm^2) within each RV or LV section were selected, and positive TP expression was determined in each field using QuPath's automatic positive pixel counting feature, where the DAB threshold was set within each staining run and applied to all sections from that experiment. Like other analyses described above, analyses on LV sections were carried out on fields from the ventricular free wall, where the septal region was excluded. The resulting mean TP positivity value for each animal was normalized to the mean of the No MCT, Sham, No SuHx, or WT control groups from each of the MCT-PAH, PAB, SuHx-PAH or *BMPR2* Δ71 animal cohorts, respectively.

Measurement of intracellular calcium ($[Ca^{2+}]_i$) mobilization

A human embryonic kidney (HEK) 293 cell line stably over-expressing a hemagglutinin (HA) epitope-tagged form of the rat TP (HEK.rTP) was generated as per previously described protocols.(9) HEK.rTP cells were routinely cultured in minimal essential medium (MEM) with Earle's salts supplemented with 10% fetal bovine serum (FBS) and maintained at 37 °C in 5 % CO_2. Prior to performing calcium mobilization assays, HEK.rTP cells were washed and harvested in Krebs-HEPES buffer (118 mM NaCl, 4.7 mM KCl, 1.2 mM $MgSO_4$, 1.2 mM KH_2PO_4, 4.2 mM $NaHCO_3$, 11.7 mM D-glucose, 1.3 mM $CaCl_2$, 10 mM HEPES, pH 7.4). Cells were then incubated with 3 µM Fluo-4 AM (ThermoFisher Scientific) in Krebs-HEPES buffer containing 1% Pluronic F-127 for 1 hour at 25 °C. The cells were washed and diluted with Krebs-HEPES buffer containing 0.5 % bovine serum albumin (BSA), to provide a final concentration of 3 x 10^5 cells/mL. Cells were then plated in black 96-well microplates at a density of approximately 50,000 cells/well (160 µL) with either vehicle (0.1 % DMSO) or with the TP antagonist, *NTP42*, where the antagonist concentration was 10X the desired concentration (0.01 nM - 100 µM) in 20 µL volume. Fluorescence measurements were performed using the Fluoroskan Ascent microplate fluorometer (ThermoFisher Scientific) based on the protocol described previously.(10) In brief, fluorescence intensity was measured at 520 nm emission wavelength (excitation wavelength 485 nm) for 20 seconds to monitor baseline prior to the addition (20 µL) of the agonist U46619, to achieve the final concentration of 1 µM. Fluorescence intensity was monitored for a further 120 seconds. Calibration of the fluorescence intensity was performed in each sample by adding 1% Triton X-100 to obtain the maximal fluorescence ratio (F_{max}) and then 300 mM EGTA to obtain the minimal fluorescence ratio (F_{min}). Intracellular Ca^{2+} ($[Ca^{2+}]_i$) mobilization was then calculated from: $[Ca^{2+}] = K_d(F - F_{min})/(F_{max} - F)$, where the equilibrium dissociation constant (K_d) for Fluo-4 AM is 345 nM. Data were expressed as the mean ± standard error of the mean (SEM) percentage of the agonist-induced response in vehicle-treated cells (Percentage of Control; %) and represent data from at least 4 independent experiments, where *NTP42* treatments were carried out in replicate within each experiment. Dose response curves were fitted using nonlinear regression and half maximal inhibitory concentration (IC_{50}) values were estimated from the curves using GraphPad Prism (Version 9, GraphPad Software).

Measurement of platelet aggregation

Approximately 5-9 mL of blood was obtained through abdominal aorta puncture from an anesthetized rat (which did not undergo any other surgery or experimental procedures) using a 19G needle attached to a syringe, using as little pulling pressure as possible. The blood was transferred into a polypropylene tube containing 3.2% sodium citrate and 10 µM indomethacin and the sample was centrifuged at 200 × *g* for 10 min at room temperature to obtain platelet-rich plasma (PRP). The residual blood was centrifuged at 2000 × *g* for 10 min to obtain the platelet-poor plasma (PPP). The PPP was diluted 1:5 in Resuspension Buffer. Platelets in PRP were counted using a hemocytometer and were diluted using the homologous PPP fraction to obtain a final count of 150,000 platelets/µL. Diluted PRP samples were transferred into Chrono-Log tubes containing a stir bar. Optical density was recorded using a Chrono-Log Aggregometer, where following the start, the recording was allowed to stabilize for approximately 30-60 s. The aliquots of PRP were then pre-incubated at 37 ± 2 °C for 10 min with either vehicle (1 % DMSO final) or *NTP42* at concentrations of 0.4, 0.5, 1, 2, 5, 10 and 20 µM, prior to initiation of aggregation using U46619 (3 µM). In the case of U46619-induced platelet aggregation, the PRP was first incubated with 20 µM indomethacin for 1 min, 100 µM of $CaCl_2$ for 30 s, followed by the addition of collagen (3 µL of 0.5 mg/mL solution); U46619 was subsequently added to induce platelet aggregation. As control for target specificity, aliquots of PRP were also pre-incubated with either vehicle (1 % DMSO final) or *NTP42* at 20 µM, prior to initiation of aggregation using ADP (50 µM). Thereafter, recording of the optical density was performed for up to 10 min or until the optical density reached a plateau. The data were expressed as the mean (± standard error of the mean; SE) percentage of the U46619-induced response in vehicle-treated platelets (Percentage of Control; %) and represent data from 3 independent experiments. A dose response curve was fitted using nonlinear regression, and the half maximal inhibitory concentration (IC_{50}) value was determined, using GraphPad Prism (Version 9, GraphPad Software) throughout.

Effect of *NTP42:KVA4* on cardiovascular parameters in conscious telemetered beagle dogs

The *in vivo* pharmacological effects of *NTP42:KVA4* on the cardiovascular system were investigated in Safety Pharmacology studies performed at Charles River Laboratories (Tranent, UK) according to Organization for Economic Co-operation and Development Principles of Good Laboratory Practice. In brief, free moving conscious male beagle dogs in their home pen with minimal disturbance were implanted with Data Sciences International (DSI, St Paul MN, USA) PhysioTel® Digital L21 radio transmitters. Using a partial Latin square design in 4 animals, cardiovascular effects were examined for up to 24 h following administration of single oral doses of the drug vehicle and 10, 50, or 450 mg/kg *NTP42:KVA4*. The parameters reported included mAP, HR, LV systolic pressure (LVSP), the derivative of LV pressure over time (LV dP/dT), QRS Duration, and QT Interval. For each parameter separately, data was statistically analyzed using a repeated measures ANOVA model with dose level, time and the interaction between dose level and time fitted as fixed effects. Animal and the interaction between animal and dose level was fitted as random effects. Pairwise comparisons with the control dose were made using Student's t-distribution, with no adjustment for multiple testing. If the interaction between dose level and time was significant at the 5% significance level, pairwise comparisons were made at each time point separately. If the interaction between dose level and time was not significant, the comparison was made for the study as a whole.

Statistical analysis

In all cases, potential outliers within each data set were identified based on the method of Interquartile Range (IQR) with Tukey fences, to include values falling outside either; (i) [Quartile 1 - 1.5*IQR] (Lower Fence), or (ii) [Quartile 3 + 1.5*IQR] (Upper Fence). Statistical analyses of differences were carried out using one- or two-way analysis of variance (ANOVA) with Holm-Šídák correction applied for multiple comparisons, or unpaired two-tailed Student's t-tests, as appropriate. Statistical analysis was performed using GraphPad Prism (Version 9, GraphPad Software) throughout. Reported values are expressed throughout as the mean ± standard error of the mean (SEM) and number of biological replicates (n), and the relevant statistical analysis methods are detailed within the corresponding figure legend. P values < 0.05 were considered to indicate statistically significant differences. In pairwise comparisons, *, **, *** and **** denote $P < 0.05$, 0.01, 0.001, and 0.0001, versus the 'MCT – Placebo' or 'PAB – Placebo' diseased control groups within the MCT-PAH or PAB studies, respectively.

SUPPLEMENTAL TABLES

Table S1 – Treatment Group Assignment and Treatment Information

Table S1A – MCT-induced model of experimental PAH

Group Number	Group Name	Disease Induction	Treatment	Dosage#	Started	Deaths$	Final Analysis
1	No MCT	Saline	Placebo*	30 mg/kg BID	6	0	6
2	MCT	MCT	Placebo*	30 mg/kg BID	14	3	11
3	*NTP42:KVA4*	MCT	*NTP42:KVA4*	1 mg/kg BID	10	2	8
4	Sildenafil	MCT	Sildenafil	50 mg/kg BID	12	1	11
5	Macitentan	MCT	Macitentan	30 mg/kg BID	12	3	9
6	Selexipag	MCT	Selexipag	1 mg/kg BID	12	0	12
7	Riociguat	MCT	Riociguat	5 mg/kg BID	12	2	10

* Placebo was 30 mg/kg Kollidon® VA 64
# All treatments were delivered as a suspension in 0.5 % (w/v) HPMC-E3 by oral gavage
$ Animal deaths that occurred following MCT injection and before terminal surgery.

Table S1B – PAB model of right heart overload

Group Number	Group Name	Surgical Procedure	Treatment	Dosage#	Started	Deaths$	Final Analysis
1	Sham	Sham	Placebo*	30 mg/kg BID	7	0	7
2	PAB	PAB	Placebo*	30 mg/kg BID	10	2	8
3	*NTP42:KVA4*	PAB	*NTP42:KVA4*	1 mg/kg BID	8	0	8
4	Riociguat	PAB	Riociguat	5 mg/kg BID	7	1	5†

* Placebo was 30 mg/kg Kollidon® VA 64
# All treatments were delivered as a suspension in 0.5 % (w/v) HPMC-E3 by oral gavage
$ Animal deaths that occurred following treatment commencement on Day 2 post-surgery and before terminal surgery
† Only animals where a peak PA pressure gradient ≥ 20 mmHg after PAB surgery was observed were included in the final analysis set. Following this criterion, one animal was excluded from the Riociguat group.

Table S2 – Human Tissue Samples from Healthy Donors and from PAH Patients and other RV Failure Pathologies

Case ID	Clinical Pathology	Gender, Age
5217*	Healthy	F, 58
5224	Healthy	F, 55
5246*	Healthy	F, 54
5285	Healthy	M, 51
5343	Healthy	F, 34
5100*	PAH	F, 57
5144*	PAH	F, 51
5118	RV Failure (Primary DCM)	M, 58
5199	RV Failure (Secondary DCM)	M, 50
5278	RV Failure (Primary DCM)	F, 23

* In addition to RV tissue, matching LV tissue was also obtained for these patient cases

Table S3 – TaqMan® Gene Expression Assays

Species	Gene	TaqMan® Assay ID*
Rat	*Col1a1* (collagen, type I, alpha 1)	Rn01463848_m1
Rat	*Col3a1* (collagen, type III, alpha 1)	Rn01437681_m1
Rat	*Nppa* (natriuretic peptide A; ANP)	Rn00664637_g1
Rat	*Nppb* (natriuretic peptide B; BNP)	Rn00580641_m1
Rat	*Tbxa2r* (thromboxane A_2 receptor; TP)	Rn00690601_m1
Rat	*Gapdh* (glyceraldehyde-3-phosphate dehydrogenase)	Rn99999916_s1
Human	*TBXA2R* (thromboxane A_2 receptor; TP)	Hs00169054_m1
Human	*GAPDH* (glyceraldehyde-3-phosphate dehydrogenase)	Hs99999905_m1

* All TaqMan® gene expression assays were obtained from ThermoFisher and used as directed.

Table S4 – Effect of Drug Treatment on Parameters in the MCT-PAH Model

Parameter	Treatment Group[†]<br>Mean ± SEM (n)[#]<br>t Test vs MCT Only – Placebo Group[$]						
	**No MCT<br>Placebo**	**MCT Only<br>Placebo**	***NTP42:KVA4*<br>1 mg/kg BID**	**Sildenafil<br>50 mg/kg BID**	**Macitentan<br>30 mg/kg BID**	**Selexipag<br>1 mg/kg BID**	**Riociguat<br>5 mg/kg BID**
**mPAP (mmHg)**	**13.9 ± 0.8 (6)<br>***, P = 0.0002**	40.5 ± 3.9 (11)	**27.6 ± 3.6 (8)<br>*, P = 0.0327**	**24.7 ± 3.1 (11)<br>**, P = 0.0047**	**26.2 ± 2.6 (9)<br>**, P = 0.0095**	36.9 ± 3.0 (12)<br>ns, P = 0.4669	**26.4 ± 3.1 (10)<br>*, P = 0.0114**
**RVSP (mmHg)**	**19.9 ± 0.8 (5)<br>***, P = 0.0004**	66.7 ± 6.7 (11)	**37.8 ± 3.3 (7)<br>**, P = 0.0050**	**40.7 ± 5.1 (11)<br>**, P = 0.0060**	**38.5 ± 3.4 (9)<br>**, P = 0.0026**	65.5 ± 6.1 (12)<br>ns, P = 0.8967	**43.5 ± 4.7 (10)<br>*, P = 0.0119**
**mAP (mmHg)**	103.7 ± 10.4 (6)<br>ns, P = 0.1976	91.9 ± 3.3 (11)	99.8 ± 7.1 (8)<br>ns, P = 0.2821	92.4 ± 2.4 (10)<br>ns, P = 0.9092	99.4 ± 3.9 (8)<br>ns, P = 0.1562	89.9 ± 5.6 (12)<br>ns, P = 0.7606	**109.3 ± 4.2 (8)<br>**, P = 0.0043**
**HR (bpm)**	308.5 ± 28.2 (6)<br>ns, P = 0.8658	304.8 ± 4.3 (11)	310.7 ± 14.7 (8)<br>ns, P = 0.6676	283.0 ± 12.9 (11)<br>ns, P = 0.1244	284.5 ± 11.5 (9)<br>ns, P = 0.0926	**280.6 ± 9.3 (11)<br>*, P = 0.0284**	315.3 ± 16.4 (10)<br>ns, P = 0.5290
**Pulmonary Vessel Occlusion (%)**	**20.0 ± 0.7 (6)<br>****, P < 0.0001**	33.4 ± 1.5 (11)	**26.6 ± 1.3 (7)<br>**, P = 0.0050**	**25.4 ± 1.2 (11)<br>***, P = 0.0004**	**27.2 ± 1.2 (9)<br>**, P = 0.0059**	**29.0 ± 0.7 (10)<br>*, P = 0.0149**	**23.9 ± 1.0 (10)<br>****, P < 0.0001**
**Fully-muscularized Pulmonary Vessels (%)**	**4.3 ± 1.6 (6)<br>****, P < 0.0001**	29.7 ± 3.1 (11)	**14.2 ± 2.8 (8)<br>**, P = 0.0022**	**18.9 ± 2.8 (11)<br>*, P = 0.0167**	**15.7 ± 2.9 (9)<br>**, P = 0.0043**	**19.4 ± 1.9 (11)<br>**, P = 0.0097**	**21.1 ± 1.7 (9)<br>*, P = 0.0339**
**$CD68^+$ Pulmonary Macrophages (Cells/mm^2)**	**78.0 ± 4.6 (5)<br>****, P < 0.0001**	493.3 ± 28.5 (11)	**112.3 ± 15.2 (8)<br>****, P < 0.0001**	**114.1 ± 22.7 (10)<br>****, P < 0.0001**	**184.3 ± 33.4 (9)<br>****, P < 0.0001**	**147.9 ± 26.9 (12)<br>****, P < 0.0001**	**146.3 ± 23.2 (9)<br>****, P < 0.0001**
**Proportion Fully-fibrotic Pulmonary Vessels (%)**	**11.2 ± 2.5 (5)<br>***, P = 0.0003**	43.2 ± 4.3 (11)	**29.8 ± 2.6 (8)<br>*, P = 0.0266**	44.6 ± 5.0 (10)<br>ns, P = 0.8231	43.1 ± 1.9 (8)<br>ns, P = 0.9880	46.3 ± 2.5 (12)<br>ns, P = 0.5209	39.7 ± 3.7 (10)<br>ns, P = 0.5473
**Lung Weight/Tibia Length Ratio (%)**	**3.98 ± 0.15 (6)<br>****, P < 0.0001**	7.12 ± 0.37 (11)	**5.51 ± 0.29 (7)<br>**, P = 0.0069**	6.57 ± 0.35 (11)<br>ns, P = 0.2937	6.87 ± 0.27 (9)<br>ns, P = 0.6145	7.03 ± 0.36 (12)<br>ns, P = 0.8593	6.59 ± 0.22 (10)<br>ns, P = 0.2481
**Fulton's Index**	**0.280 ± 0.004 (5)<br>****, P < 0.0001**	0.594 ± 0.018 (9)	**0.442 ± 0.039 (8)<br>**, P = 0.0023**	**0.473 ± 0.041 (11)<br>*, P = 0.0225**	**0.466 ± 0.024 (8)<br>***, P = 0.0006**	**0.667 ± 0.017 (10)<br>**, P = 0.0094**	**0.488 ± 0.034 (10)<br>*, P = 0.0174**
**RV Cardiomyocyte Size (Cross-sectional Area, μm^2)**	**190.8 ± 8.9 (5)<br>****, P < 0.0001**	300.0 ± 9.9 (11)	**259.3 ± 12.3 (8)<br>*, P = 0.0184**	269.7 ± 15.5 (11)<br>ns, P = 0.1149	303.2 ± 13.0 (8)<br>ns, P = 0.8435	**339.1 ± 13.4 (12)<br>*, P = 0.0309**	273.2 ± 9.4 (10)<br>ns, P = 0.0649
**RV Vascularization (Capillaries/mm^2)**	**3701 ± 48 (5)<br>**, P = 0.0013**	2878 ± 134 (11)	3038 ± 148 (8)<br>ns, P = 0.4407	**3920 ± 325 (11)<br>**, P = 0.0077**	2946 ± 229 (8)<br>ns, P = 0.7904	3187 ± 238 (12)<br>ns, P = 0.2837	3200 ± 75 (7)<br>ns, P = 0.0930

**RV Adaptation Index**	**1.00 ± 0.07 (6)<br>****, P < 0.0001**	0.53 ± 0.03 (11)	**0.66 ± 0.06 (8)<br>*, P = 0.0390**	**0.83 ± 0.09 (11)<br>**, P = 0.0035**	0.58 ± 0.07 (9)<br>ns, P = 0.5282	0.51 ± 0.03 (12)<br>ns, P = 0.7502	**0.68 ± 0.05 (9)<br>*, P = 0.0135**
**RV Fibrosis (Fold Change)**	**1.0 ± 0.0 (6)<br>***, P = 0.0002**	2.8 ± 0.3 (11)	**1.5 ± 0.1 (7)<br>**, P = 0.0016**	**1.9 ± 0.2 (11)<br>*, P = 0.0149**	**1.8 ± 0.1 (8)<br>**, P = 0.0060**	3.0 ± 0.1 (10)<br>ns, P = 0.5929	**1.8 ± 0.2 (10)<br>**, P = 0.0041**
**RV TP Expression (Fold Change)**	**1.00 ± 0.05 (5)<br>***, P = 0.0002**	2.29 ± 0.17 (11)	1.97 ± 0.22 (8)<br>ns, P = 0.2575	2.60 ± 0.09 (10)<br>ns, P = 0.1389	2.40 ± 0.12 (8)<br>ns, P = 0.6237	2.31 ± 0.18 (12)<br>ns, P = 0.9251	2.70 ± 0.23 (9)<br>ns, P = 0.1645
**Liver Weight/Body Weight Index (%)**	3.24 ± 0.12 (6)<br>ns, P = 0.8740	3.26 ± 0.09 (11)	3.21 ± 0.10 (7)<br>ns, P = 0.7064	**3.73 ± 0.12 (11)<br>**, P = 0.0041**	**3.85 ± 0.15 (9)<br>**, P = 0.0028**	3.21 ± 0.12 (12)<br>ns, P = 0.7039	**3.89 ± 0.12 (10)<br>***, P = 0.0004**

Abbreviations: MCT, monocrotaline; SEM, standard error of the mean; mPAP, mean pulmonary arterial pressure; RVSP, right ventricular systolic pressure; mAP, mean systemic arterial pressure; HR, heart rate; RV, right ventricle

† All treatments were delivered as a suspension in 0.5 % (w/v) HPMC-E3 by oral gavage, ‡ Placebo was 30 mg/kg Kollidon® VA 64

# The numbers (n) given in this table refer to the number of input data used for the individual experimental parameter following removal of any justifiable outliers identified using the method of IQR with Tukey fences. **Table S1A** provides details on numbers of animals enrolled into the studies and those that survived through to terminal surgery.

$ Unpaired Student's t test versus the 'MCT Only – Placebo' control group, where *, **, *** and **** denote P < 0.05, 0.01, 0.001, and 0.0001; ns, not significant.

Table S5 – Effect of Drug Treatment on Parameters in the PAB Model

**Parameter**	**Treatment Group†<br>Mean ± SEM (n)#<br>t Test vs PAB – Placebo Group$**			
	**Sham<br>Placebo**	**PAB<br>Placebo**	***NTP42:KVA4*<br>1 mg/kg BID**	**Riociguat<br>5 mg/kg BID**
**RVFWT (mm)**	**0.59 ± 0.03 (6)<br>****, P < 0.0001**	1.70 ± 0.05 (8)	1.73 ± 0.06 (7)<br>ns, P = 0.6749	1.75 ± 0.01 (4)<br>ns, P = 0.4992
**RVEDD (mm)**	**5.52 ± 0.19 (6)<br>**, P = 0.0014**	7.06 ± 0.29 (8)	**6.08 ± 0.31 (8)<br>*, P = 0.0349**	7.09 ± 0.21 (4)<br>ns, P = 0.9556
**RVEDA (cm^2)**	**0.23 ± 0.01 (7)<br>***, P = 0.0008**	0.39 ± 0.03 (8)	**0.29 ± 0.02 (7)<br>*, P = 0.0264**	0.37 ± 0.01 (3)<br>ns, P = 0.7628
**RAA (cm^2)**	**0.25 ± 0.02 (6)<br>***, P = 0.0007**	0.45 ± 0.03 (8)	**0.34 ± 0.03 (8)<br>*, P = 0.0364**	0.47 ± 0.06 (5)<br>ns, P = 0.7373
**HR (bpm)**	**376.64 ± 8.60 (5)<br>***, P = 0.0006**	321.08 ± 7.58 (8)	331.66 ± 7.13 (7)<br>ns, P = 0.3318	332.40 ± 22.53 (5)<br>ns, P = 0.5802

**mAP (mmHg)**	104.66 ± 4.51 (6)<br>ns, P = 0.9054	103.62 ± 6.53 (8)	106.41 ± 3.24 (7)<br>ns, P = 0.7209	120.81 ± 2.36 (4)<br>ns, P = 0.1034
**ESP (mmHg)**	**21.04 ± 0.54 (5)<br>****, P < 0.0001**	86.03 ± 6.85 (8)	85.71 ± 9.40 (8)<br>ns, P = 0.9786	77.00 ± 4.78 (4)<br>ns, P = 0.4071
**EDP (mmHg)**	**2.37 ± 0.21 (6)<br>**, P = 0.0081**	4.80 ± 0.64 (8)	4.20 ± 0.49 (8)<br>ns, P = 0.4667	4.68 ± 0.87 (5)<br>ns, P = 0.9112
**ESV (µL)**	**112.13 ± 11.48 (6)<br>***, P = 0.0002**	247.71 ± 20.25 (8)	**159.09 ± 25.34 (8)<br>*, P = 0.0162**	**186.60 ± 11.41 (5)<br>*, P = 0.0480**
**EDV (µL)**	**259.22 ± 23.89 (6)<br>**, P = 0.0026**	372.95 ± 18.94 (8)	303.76 ± 28.80 (8)<br>ns, P = 0.0645	**295.94 ± 31.40 (5)<br>*, P = 0.0463**
**CO (mL/min)**	66.92 ± 5.95 (6)<br>ns, P = 0.0929	54.82 ± 3.65 (8)	57.94 ± 5.16 (8)<br>ns, P = 0.6287	51.60 ± 0.92 (3)<br>ns, P = 0.6152
**RV EF (%)**	**76.43 ± 5.48 (6)<br>***, P = 0.0003**	47.68 ± 2.29 (7)	**71.56 ± 5.18 (8)<br>**, P = 0.0015**	**62.19 ± 3.69 (5)<br>**, P = 0.0055**
**Ees (mmHg/µL)**	**0.09 ± 0.02 (6)<br>****, P < 0.0001**	0.41 ± 0.04 (8)	0.42 ± 0.07 (7)<br>ns, P = 0.8305	0.32 ± 0.02 (4)<br>ns, P = 0.1378
**Eed (mmHg/µL)**	**0.006 ± 0.001 (6)<br>*, P = 0.0164**	0.010 ± 0.001 (8)	0.007 ± 0.001 (7)<br>ns, P = 0.0913	0.012 ± 0.002 (5)<br>ns, P = 0.2908
**Fulton's Index**	**0.25 ± 0.00 (6)<br>****, P < 0.0001**	0.60 ± 0.02 (8)	0.57 ± 0.02 (7)<br>ns, P = 0.2042	0.58 ± 0.00 (4)<br>ns, P = 0.4036
**RV Cardiomyocyte Size<br>(Cross-sectional Area, μm^2)**	**283.36 ± 16.04 (7)<br>****, P < 0.0001**	610.01 ± 26.56 (8)	**475.57 ± 7.98 (7)<br>***, P = 0.0005**	598.64 ± 4.71 (4)<br>ns, P = 0.7744
**RV Vascularization<br>(Capillaries/mm^2)**	**2915 ± 97 (6)<br>****, P < 0.0001**	1865 ± 54 (7)	**2395 ± 105 (8)<br>***, P = 0.0009**	2021 ± 68 (5)<br>ns, P = 0.1013
**RV Adaptation Index**	**1.00 ± 0.11 (7)<br>****, P < 0.0001**	0.29 ± 0.02 (8)	**0.44 ± 0.03 (8)<br>***, P = 0.0006**	0.31 ± 0.01 (5)<br>ns, P = 0.4270
**ANP mRNA (Relative Expression)**	**1.05 ± 0.14 (7)<br>****, P < 0.0001**	336.05 ± 47.58 (8)	**188.21 ± 21.66 (7)<br>*, P = 0.0185**	254.54 ± 41.45 (5)<br>ns, P = 0.2624
**BNP mRNA (Relative Expression)**	**1.04 ± 0.12 (7)<br>****, P < 0.0001**	8.56 ± 0.30 (8)	7.56 ± 0.98 (8)<br>ns, P = 0.3468	7.11 ± 1.06 (5)<br>ns, P = 0.1371
**RV Fibrosis (Fold Change)**	**1.00 ± 0.06 (7)<br>***, P = 0.0001**	1.50 ± 0.06 (8)	1.42 ± 0.08 (8)<br>ns, P = 0.4662	1.80 ± 0.21 (5)<br>ns, P = 0.1282

**Collagen I/III Ratio**	**1.00 ± 0.01 (7)<br>*, P = 0.0181**	1.27 ± 0.09 (8)	**0.95 ± 0.06 (6)<br>*, P = 0.0183**	**0.92 ± 0.06 (4)<br>*, P = 0.0295**
**Passive Tension, at 2.2 µm Sarcomere Length (mN/mm^2)**	**1.95 ± 0.24 (6)<br>***, P = 0.0018**	4.15 ± 0.47 (6)	**1.74 ± 0.25 (6)<br>***, P = 0.0010**	4.87 ± 0.46 (5)<br>ns, P = 0.3027
**Maximum Active Tension (mN/mm^2)**	18.60 ± 3.08 (6)<br>ns, P = 0.1816	26.11 ± 4.23 (6)	22.69 ± 1.39 (6)<br>ns, P = 0.4596	27.35 ± 2.81 (5)<br>ns, P = 0.8212
**Ca^{2+} Sensitivity, pCa_{50}**	5.63 ± 0.03 (6)<br>ns, P = 0.3916	5.67 ± 0.03 (6)	5.65 ± 0.03 (6)<br>ns, P = 0.7815	5.69 ± 0.03 (5)<br>ns, P = 0.6232
**NCX1 Full Length Protein (Fold Change)**	1.00 ± 0.05 (7)<br>ns, P = 0.5459	1.07 ± 0.10 (7)	1.16 ± 0.12 (8)<br>ns, P = 0.5711	1.13 ± 0.19 (5)<br>ns, P = 0.7797
**NCX1 Degraded Fragment (% of Total NCX1)**	**4.94 ± 0.87 (7)<br>****, P < 0.0001**	15.54 ± 1.46 (7)	**10.27 ± 1.06 (6)<br>*, P = 0.0164**	15.04 ± 3.30 (5)<br>ns, P = 0.8794
**SERCA2A Full Length Protein (Fold Change)**	**1.00 ± 0.03 (7)<br>**, P = 0.0035**	0.85 ± 0.03 (8)	0.82 ± 0.03 (8)<br>ns, P = 0.5325	0.88 ± 0.03 (5)<br>ns, P = 0.5097
**SERCA2A Degraded Fragment (% of Total SERCA2A)**	**3.78 ± 0.97 (6)<br>****, P < 0.0001**	19.97 ± 2.18 (8)	14.47 ± 1.65 (8)<br>ns, P = 0.0638	13.72 ± 3.84 (5)<br>ns, P = 0.1533
**RV TP Expression (Fold Change)**	**1.00 ± 0.31 (7)<br>*, P = 0.0218**	2.62 ± 0.51 (8)	1.59 ± 0.36 (7)<br>ns, P = 0.1311	2.28 ± 0.57 (5)<br>ns, P = 0.6771
**Liver Weight/Body Weight Index (%)**	3.48 ± 0.16 (7)<br>ns, P = 0.2711	3.29 ± 0.06 (7)	3.27 ± 0.09 (8)<br>ns, P = 0.9151	**3.80 ± 0.16 (5)<br>**, P = 0.0055**

Abbreviations: PAB, pulmonary arterial banding; SEM, standard error of the mean; RVFWT, RV free wall thickness at diastole; RVEDD, RV end-diastolic dimension; RVEDA, RV end-diastolic area; RAA, maximum right atrial area; HR, heart rate; mAP, mean systemic arterial pressure; ESP, RV end-systolic pressure; EDP, RV end-diastolic pressure; ESV, RV end-systolic volume; EDV, RV end-diastolic volume; CO, cardiac output; RV EF, RV ejection fraction; Ees, end-systolic elastance; Eed, end-diastolic elastance; NCX1, Na+/Ca2+ exchanger 1; SERCA2A, sarco/endoplasmic reticulum Ca2+-ATPase 2A; RV, right ventricle

[†] All treatments were delivered as a suspension in 0.5 % (w/v) HPMC-E3 by oral gavage, [‡] Placebo was 30 mg/kg Kollidon® VA 64

[#] The numbers (n) given in this table refer to the number of input data used for the individual experimental parameter following removal of any justifiable outliers identified using the method of IQR with Tukey fences. **Table S1B** provides details on numbers of animals enrolled into the studies and those that survived through to terminal surgery.

[$] Unpaired Student's t test versus the 'PAB – Placebo' control group, where *, **, *** and **** denote P < 0.05, 0.01, 0.001, and 0.0001; ns, not significant.

SUPPLEMENTAL REFERENCES

1. Mulvaney EP, Reid HM, Bialesova L, Mendes-Ferreira P, Adao R, Bras-Silva C, et al. Efficacy of the Thromboxane Receptor Antagonist Ntp42 Alone, or in Combination with Sildenafil, in the Sugen/Hypoxia-Induced Model of Pulmonary Arterial Hypertension. *Eur J Pharmacol* (2020) 889:173658. Epub 2020/10/31. doi: 10.1016/j.ejphar.2020.173658.
2. Bankhead P, Loughrey MB, Fernandez JA, Dombrowski Y, McArt DG, Dunne PD, et al. Qupath: Open Source Software for Digital Pathology Image Analysis. *Sci Rep* (2017) 7(1):16878. Epub 2017/12/06. doi: 10.1038/s41598-017-17204-5.
3. Schindelin J, Rueden CT, Hiner MC, Eliceiri KW. The Imagej Ecosystem: An Open Platform for Biomedical Image Analysis. *Molecular reproduction and development* (2015) 82(7-8):518-29. Epub 2015/07/15. doi: 10.1002/mrd.22489.
4. Ruifrok AC, Johnston DA. Quantification of Histochemical Staining by Color Deconvolution. *Anal Quant Cytol Histol* (2001) 23(4):291-9. Epub 2001/09/04.
5. Mendes-Ferreira P, Santos-Ribeiro D, Adao R, Maia-Rocha C, Mendes-Ferreira M, Sousa-Mendes C, et al. Distinct Right Ventricle Remodeling in Response to Pressure Overload in the Rat. *Am J Physiol Heart Circ Physiol* (2016) 311(1):H85-95. Epub 2016/05/21. doi: 10.1152/ajpheart.00089.2016.
6. Goncalves-Rodrigues P, Almeida-Coelho J, Goncalves A, Amorim F, Leite-Moreira AF, Stienen GJM, et al. In Vitro Assessment of Cardiac Function Using Skinned Cardiomyocytes. *J Vis Exp* (2020) (160). Epub 2020/07/07. doi: 10.3791/60427.
7. Hautefort A, Mendes-Ferreira P, Sabourin J, Manaud G, Bertero T, Rucker-Martin C, et al. Bmpr2 Mutant Rats Develop Pulmonary and Cardiac Characteristics of Pulmonary Arterial Hypertension. *Circulation* (2019) 139(7):932-48. Epub 2018/12/28. doi: 10.1161/CIRCULATIONAHA.118.033744.
8. Hamdani N, Franssen C, Lourenco A, Falcao-Pires I, Fontoura D, Leite S, et al. Myocardial Titin Hypophosphorylation Importantly Contributes to Heart Failure with Preserved Ejection Fraction in a Rat Metabolic Risk Model. *Circ Heart Fail* (2013) 6(6):1239-49. Epub 2013/09/10. doi: 10.1161/CIRCHEARTFAILURE.113.000539.
9. Walsh MT, Foley JF, Kinsella BT. The Alpha, but Not the Beta, Isoform of the Human Thromboxane A2 Receptor Is a Target for Prostacyclin-Mediated Desensitization. *J Biol Chem* (2000) 275(27):20412-23.
10. Kassack MU. Quantitative Comparison of Functional Screening by Measuring Intracellular Ca2+ with Radioligand Binding at Recombinant Human Dopamine Receptors. *AAPS PharmSci* (2002) 4(4):E31. Epub 2003/03/21. doi: 10.1208/ps040431.

SUPPLEMENTAL FIGURES

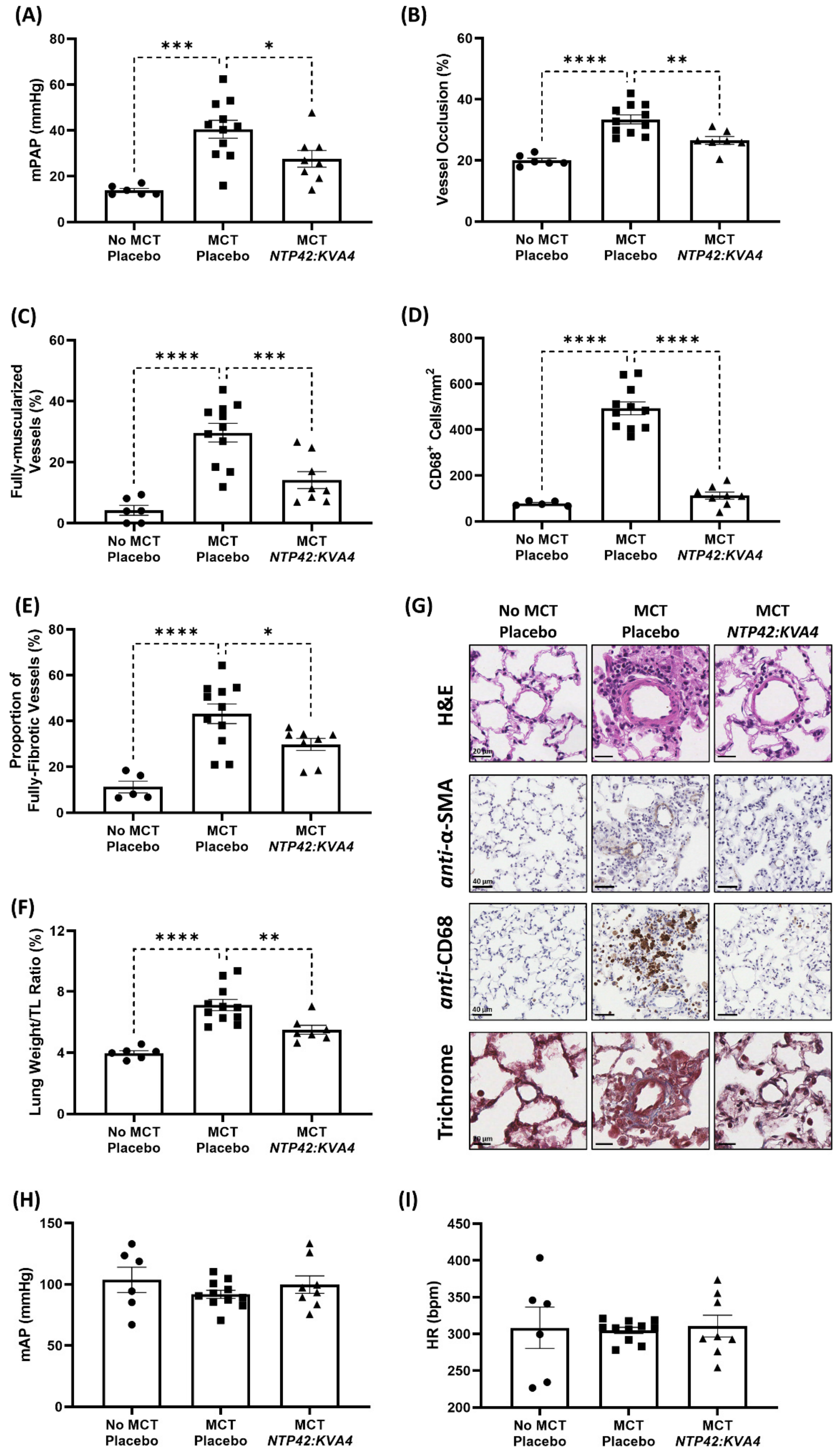

Figure S1: Effect of *NTP42:KVA4* Treatment on Hemodynamics and Pulmonary Pathology in the MCT-PAH Model

Panels A-E show hemodynamic measurements of: **(A)** mPAP in the 'No MCT - Placebo', 'MCT – Placebo', and *NTP42:KVA4* groups [n = 6, 11, and 8, respectively]; **(B)** Degree of pulmonary vessel occlusion [n = 6, 11, and 7, respectively]; **(C)** Proportion of fully-muscularized pulmonary vessels [n = 6, 11, 8]; **(D)** Pulmonary $CD68^+$ macrophage density [n = 5, 11, 8]; **(E)** Proportion of fully-fibrotic pulmonary vessels [n = 5, 11, 8], and **(F)** Index of pulmonary edema, calculated as a ratio of total lung weight (g) by the tibia length (TL, mm) for each animal, and expressed as a percentage [n = 6, 11, 7]. Panel **(G)** shows representative photomicrographs, selected from a random animal from each treatment group, of: Hematoxylin and eosin (H&E)-stained lung tissue captured at 600× magnification (scale bars, 20 µm); *Anti*-α-smooth muscle actin (SMA)-stained lung tissue captured at 250× magnification (scale bars, 40 µm); *Anti*-CD68-stained lung tissue captured at 250× magnification (scale bars, 40 µm), and Masson's trichrome-stained lung tissue captured at 400× magnification (scale bars, 20 µm). Panels H-I show hemodynamic measurements of: **(H)** mAP [n = 6, 11, 8], and **(I)** HR [n = 6, 11, 8]. Data presented are the mean ± SEM. * $P < 0.05$, ** $P < 0.01$, *** $P < 0.001$, **** $P < 0.0001$ *vs.* 'MCT – Placebo', according to one-way analysis of variance (ANOVA) with Holm-Šídák correction applied for multiple comparisons. Note that while **Table S1** provides details on numbers of animals enrolled into the studies reported herein and those that survived through to terminal surgery, the numbers (n) given in the square brackets in all figure legends refer to the number of input data used for the individual experimental parameter following removal of any justifiable outliers identified using the method of IQR with Tukey fences.

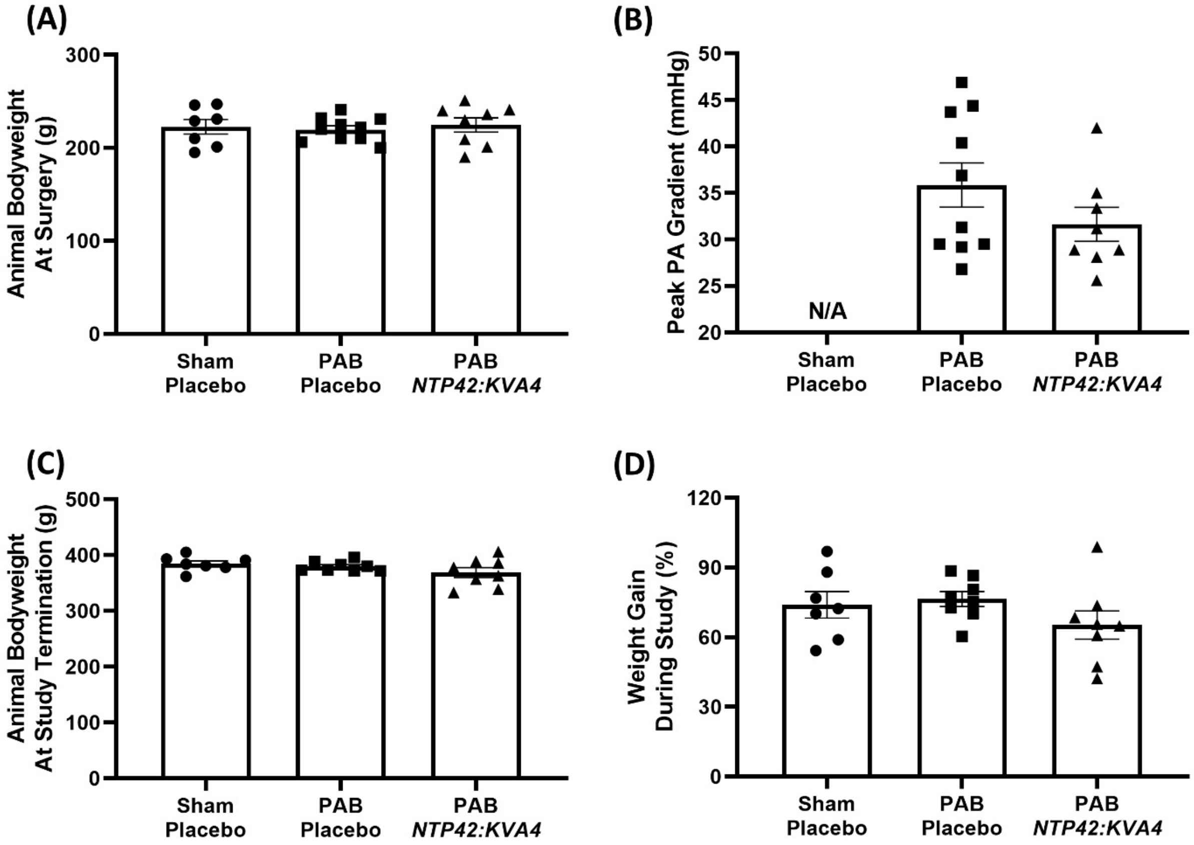

Figure S2: Animal Randomization, Pre-treatment ECHO, and Terminal Bodyweight and Weight Gain in the PAB Model

Panels A & B show: **(A)** Randomization of animals according to bodyweight was performed prior to PAB or sham surgery, where no significant difference in bodyweight distribution was observed (one-way ANOVA), and **(B)** ECHO assessment of the pressure gradient across the PA constriction performed immediately following PAB surgery, where no significant difference in peak PA pressure gradient was observed among the groups (one-way ANOVA). Panels C & D show: **(C)** Animal bodyweight at study termination, and **(B)** Percentage bodyweight gained during the course of the study, where no significant differences in terminal bodyweight or bodyweight gain were observed among the groups (one-way ANOVA).

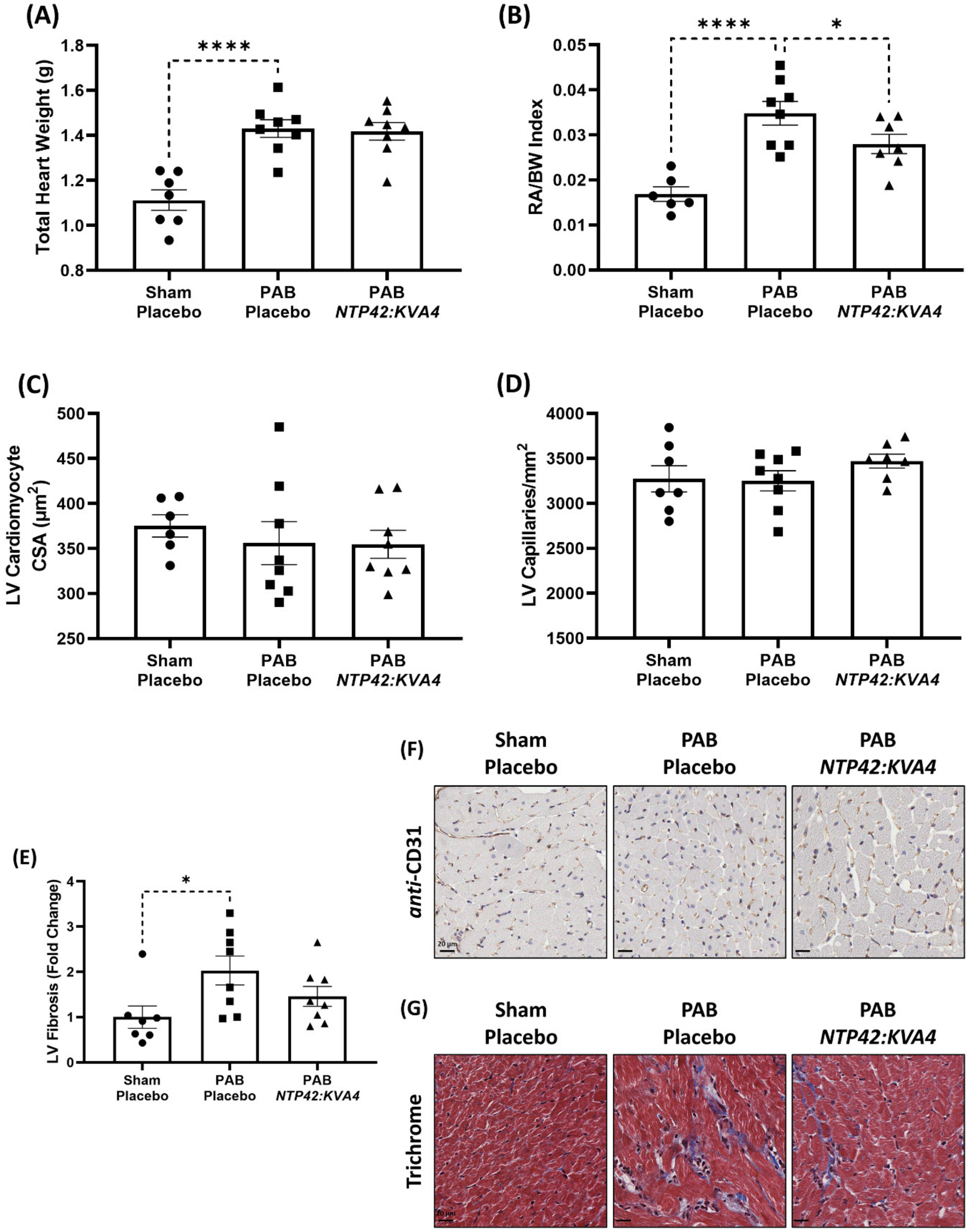

Figure S3: Effect of *NTP42:KVA4* Treatment on Total Heart Weight, RA Wall Weight, and LV Pathology in the PAB Model

Panels A-E show: **(A)** Total heart weight in the 'Sham – Placebo', 'PAB – Placebo', and *NTP42:KVA4* groups [n = 7, 8, and 8, respectively]; **(B)** RA wall weight normalized to animal bodyweight (BW) [n = 6, 8, and 7, respectively]; **(C)** LV cardiomyocyte size [n = 6, 8, and 8, respectively]; **(D)** LV vascularization [n = 7, 8, and 7, respectively], and **(E)** LV fibrosis [n = 6,

8, and 8, respectively]. Data presented are the mean ± SEM. * $P < 0.05$, **** $P < 0.0001$ *vs.* 'PAB – Placebo', according to one-way ANOVA with Holm-Šídák correction. Panels F & G show representative photomicrographs, selected from a random animal from each treatment group, of: **(F)** *Anti*-CD31-stained LV tissue captured at 400× magnification (scale bars represent 20 µm), and **(G)** Masson's trichrome-stained LV tissue captured at 400× magnification (scale bars, 20 µm).

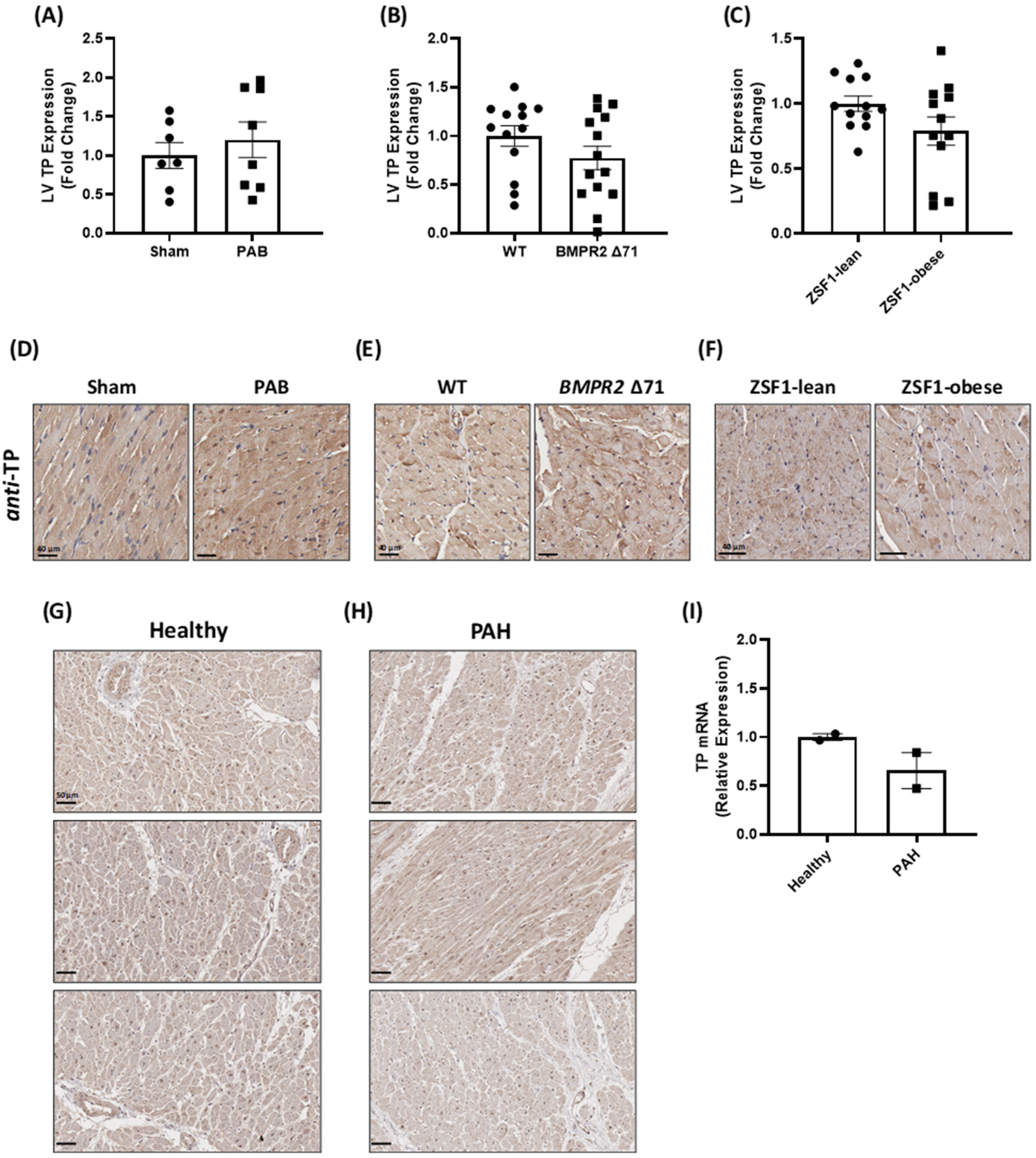

Figure S4: LV Expression of the TP in the PAB Model, *BMPR2* Δ71 & ZSF1 Rats, and Human PAH

Panels A-C show relative expression levels of the TP in LV tissue obtained from: **(A)** The PAB model, from animals of the ‘Sham’, and ‘PAB’ groups (n = 7 and 8, respectively); **(B)** WT and *BMPR2* Δ 71 animals [n = 13 and 14, respectively], and **(C)** ZSF1-lean and ZSF1-obese rats [n = 12 and 12, respectively] where no significant differences in LV TP expression was observed in each case (unpaired Student’s t tests). Data presented are the mean ± SEM. Panels D-F show representative photomicrographs, selected from a random animal from each treatment group, of *anti*-TP-stained LV tissue from: **(D)** ‘No SuHx’ and ‘SuHx’ animals; **(E)** WT or *BMPR2* Δ71 animals, and **(F)** ZSF1-lean or ZSF1-obese animals, where all images were captured at

400× magnification (scale bars represent 40 µm). Panels G-H show three representative photomicrographs of *anti*-TP-stained human RV tissue obtained from: **(G)** Healthy donors, and **(H)** PAH patients, where all images were captured at 150× magnification (scale bars represent 50 µm). Panel **(I)** shows relative LV TP mRNA expression levels in Healthy or PAH patients (n = 2 each, see **Table S2**), where no significant differences in LV TP expression was observed (unpaired Student's t test).

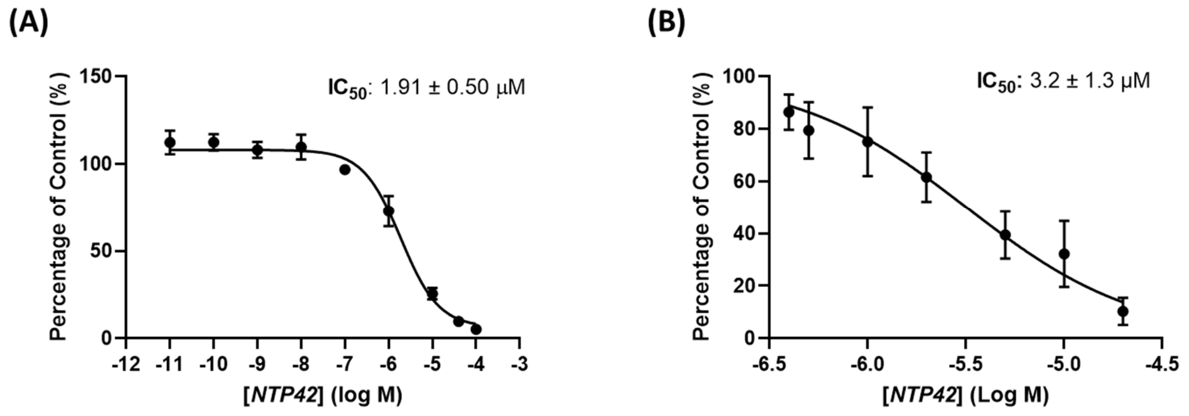

Figure S5: Effect of *NTP42* on U46619-induced Intracellular Calcium Mobilization in HEK.rTP Cells and on U46619-induced Platelet Aggregation in Rats

(A) HEK.rTP cells, preloaded with Fluo-4, were incubated with *NTP42* (0.01 nM – 100 µM), prior to stimulation with 1 µM U46619. Dose inhibition curves show the effect of *NTP42* on intracellular Ca^{2+} mobilization following stimulation with U46619. Data is presented as the mean (± SEM) percentage of the U46619-induced response in vehicle-treated cells (Percentage of Control; %); data given is representative data from at least 4 independent experiments. Calculated IC_{50} values for *NTP42* are shown alongside the dose inhibition curve. (B) Dose inhibition curve showing the effect of *NTP42* (0.4 – 20 µM) on rat platelet aggregation following stimulation with 3 µM U46619. Data is presented as the mean (± SEM) changes in percentage aggregation (Percentage of Control; %) and represents data from at least 3 independent experiments.

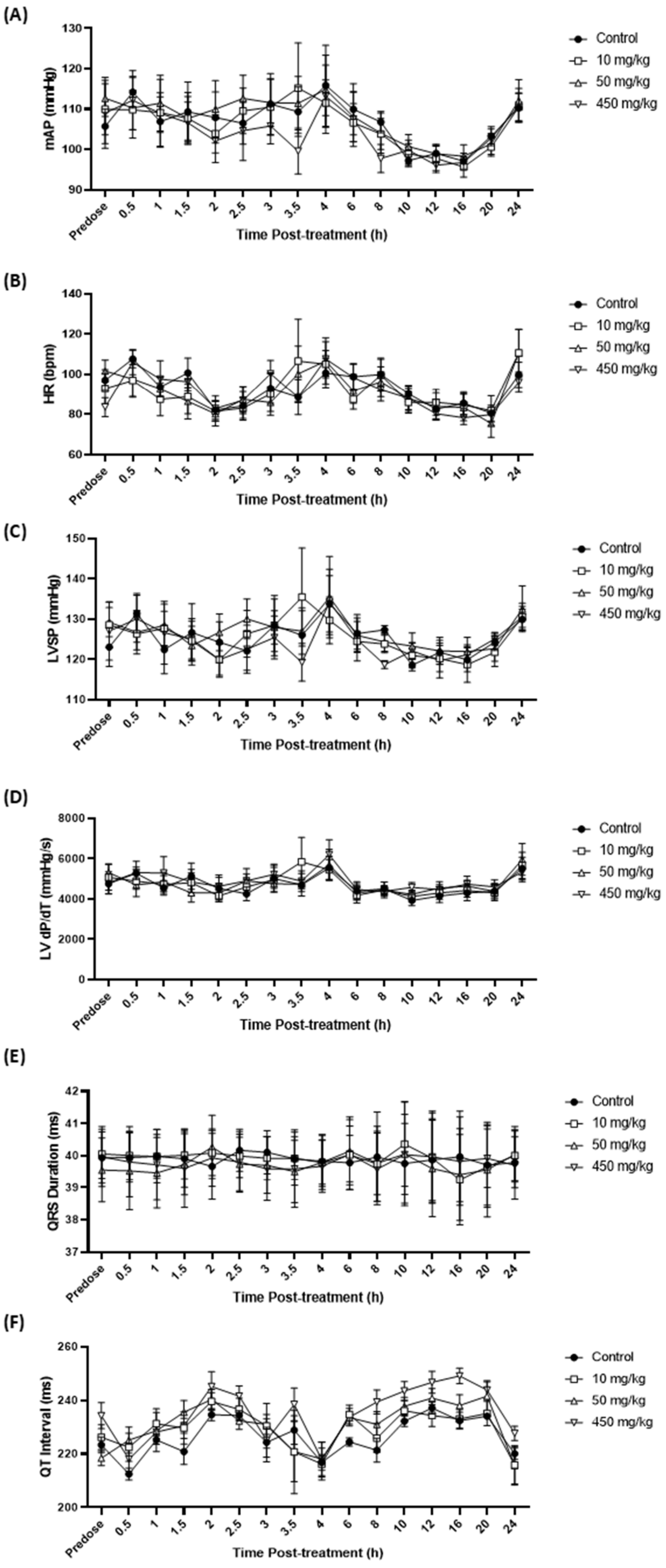
(A)
mAP (mmHg)
Time Post-treatment (h)
Control
10 mg/kg
50 mg/kg
450 mg/kg
(B)
HR (bpm)
Time Post-treatment (h)
Control
10 mg/kg
50 mg/kg
450 mg/kg
(C)
LVSP (mmHg)
Time Post-treatment (h)
Control
10 mg/kg
50 mg/kg
450 mg/kg
(D)
LV dP/dT (mmHg/s)
Time Post-treatment (h)
Control
10 mg/kg
50 mg/kg
450 mg/kg
(E)
QRS Duration (ms)
Time Post-treatment (h)
Control
10 mg/kg
50 mg/kg
450 mg/kg
(F)
QT Interval (ms)
Time Post-treatment (h)
Control
10 mg/kg
50 mg/kg
450 mg/kg
Predose
0.5
1
1.5
2
2.5
3
3.5
4
6
8
10
12
16
20
24

Figure S6: Effect of *NTP42:KVA4* on Cardiovascular Parameters in Conscious Telemetered Beagle Dogs Following Single Oral Administration

Panels A-F show the effect of *NTP42:KVA4,* after single oral (gavage) administration to conscious telemetered beagle dogs (n = 4), on: **(A)** mAP; **(B)** HR; **(C)** LVSP; **(D)** LV dP/dT; **(E)** QRS Duration, and **(F)** Measured QT Interval, where animals received drug vehicle (Control) and 10, 50, or 450 mg/kg *NTP42:KVA4* in a partial Latin square design, and where cardiovascular effects were examined for up to 24 h. Data presented are the mean ± SEM. No *NTP42:KVA4*-related effects on these measured parameters were observed compared to the time-matched Control.
